# Human milk oligosaccharide metabolism and antibiotic resistance in early gut colonizers: insights from bifidobacteria and lactobacilli in the maternal-infant microbiome

**DOI:** 10.1080/19490976.2025.2501192

**Published:** 2025-05-09

**Authors:** Anna Samarra, Simone Renwick, Aleksandr A. Arzamasov, Dmitry A. Rodionov, Kennedy Spann, Raul Cabrera-Rubio, Antia Acuna-Gonzalez, Cecilia Martínez-Costa, Lindsay Hall, Nicola Segata, Andrei L. Osterman, Lars Bode, Maria Carmen Collado

**Affiliations:** a Department of Biotechnology, Institute of Agrochemistry and Food Technology- National Spanish Research Council (IATA-CSIC), Valencia, Spain; b Department of Pediatrics, School of Medicine, University of California San Diego, La Jolla, CA, USA; c Mother-Milk-Infant Center of Research Excellence, University of California San Diego, La Jolla, CA, USA; d Infectious and Inflammatory Disease Center, Sanford Burnham Prebys Medical Discovery Institute, La Jolla, CA, USA; e Food, Microbiome and Health, Quadram Institute Bioscience, Norwich Research Park, Norwich, UK; f Department of Pediatrics, School of Medicine, University of Valencia, Valencia, Spain; g Pediatric Gastroenterology and Nutrition Section, Hospital Clínico Universitario Valencia, Valencia, Spain; h Department of Microbes, Infection and Microbiomes, School of Infection, Inflammation and Immunology, College of Medicine and Health, University of Birmingham, Birmingham, UK; i Norwich Medical School, University of East Anglia, Norwich, UK; j Department CIBIO, University of Trento, Trento, Italy; k Human Milk Institute, University of California San Diego, La Jolla, CA, USA

**Keywords:** Human milk, oligosaccharides, antibiotic resistance, bifidobacteria, infant, mother

## Abstract

Breast milk, rich in human milk oligosaccharides (HMOs), supports the early-life colonization of beneficial bacteria such as bifidobacteria and lactobacilli, potentially reducing early-life antibiotic resistance. However, antibiotic treatment may interfere with the beneficial functions of HMO-degrading bacteria. This study investigated the metabolism of HMOs by bifidobacteria and lactobacilli isolated from human milk and mother-infant paired fecal samples, along with their antibiotic resistance profiles. Understanding these species- and sample-type-specific interactions will provide valuable insights into how bioactive components in human milk may shape the infant resistome during early life. A total of 39 *Bifidobacterium* and 14 *Lactobacillaceae* strains were isolated from paired mother-infant fecal and breast milk samples. Whole genome sequencing (WGS) allowed functional predictions on the HMO metabolism abilities and the resistance genotype of each strain. *In vitro* HMO utilization was assessed using growth kinetics assays combined with HMO glycoprofiling in culture supernatant. The minimum inhibitory concentration (MIC) was also determined for each strain. HMO metabolism by the bifidobacteria was species-specific. *Bifidobacterium bifidum* (*B. bifidum*) and *Bifidobacterium longum* subsp. *infantis* (*B. infantis*) exhibited the highest capacity for HMO degradation, consistent with genomic predictions. In contrast, lactobacilli were unable to degrade HMOs *in vitro* but were predicted to metabolize the by-products of HMO degradation. Phenotypic analysis revealed that *B. bifidum* strains had the lowest levels of antibiotic resistance, while *Bifidobacterium animalis* subsp. *lactis* (*B. lactis*) strains were resistant to most tested antibiotics. Overall, *B. bifidum* demonstrated the strongest HMO-degrading ability while remaining the most antibiotic-susceptible species. Early-life colonizing bifidobacterial species possess the essential machinery required to degrade HMOs and are highly susceptible to antibiotics. A better understanding of these dynamics could inform clinical strategies to protect and restore the infant gut microbiome, particularly in neonates exposed to antibiotics.

## GRAPHICAL ABSTRACT

**Figure:**
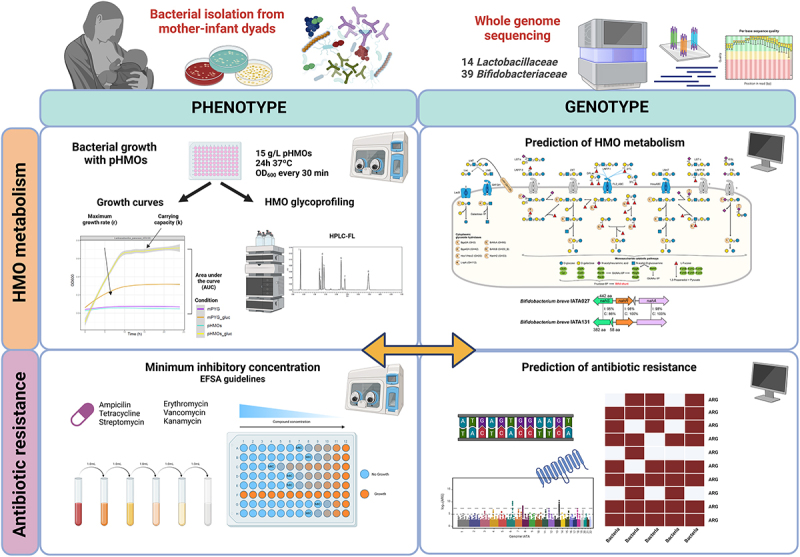

## Introduction

Human gut microbial colonization begins at birth when the neonate is exposed to microorganisms of the environment, primarily from maternal sources, which serve as the main microbial inoculum.^1,2^ The establishment and development of the microbiota occurs in parallel with other maturation processes and is modulated by external factors to which the infant is exposed during the early stages of life.^3^ Among these, mode of birth is a widely known determinant of the infant gut microbiota.^4–6^ Additionally, antibiotic use during pregnancy, delivery, or following a Cesarean (c)-section can further disrupt maternal microbial transmission.^7^ Antibiotic therapy in early life can alter microbiome diversity and disrupt microbiota-host crosstalk, resulting in microbial imbalances that can lead to short- and long-term health consequences.^8^ Furthermore, the abundance of microorganisms carrying antibiotic resistance genes (ARGs) increases in the infant’s gut following exposure to antibiotics, which has been linked to an increased risk of early-life infections.^9^ The threat of antibiotic-resistant microorganisms is especially severe in newborns, whose underdeveloped gut microbiota and immune systems make them more vulnerable than adults. Globally, antibiotic resistance is responsible for approximately 214,000 neonatal deaths annually due to septic infections, underscoring its grave effects on infant health.^10^ In early life, antibiotic resistance may emerge through multiple pathways, including horizontal gene transfer among bacteria, vertical transmission from the mother during pregnancy, childbirth, and breastfeeding, or direct exposure to antibiotics in infants.^11^ In this context, external factors to which the infant is exposed not only influence their microbiome, but also affect their resistome, which is defined as the set of ARGs carried by a population of bacteria.^3^

Breastfeeding plays a crucial role in shaping the infant’s microbiome and resistome.^12^ Human milk provides personalized nutrition during the first months of a baby’s life through a wide range of bioactive compounds that promote infant health and mold the developing infant’s microbiome.^13^ In addition, human milk also serves as a source of commensal and potentially probiotic bacteria for the newborn’s gut, with *Staphylococcus and Streptococcus* being the most dominant genera found in human milk, followed by members of the families *Lactobacillaceae* and *Bifidobacteriaceae*.^14,15^ These latter families are highly prevalent in the gut of breastfed infants due to their ability to utilize the prebiotic substrates found in human milk, such as human milk oligosaccharides (HMOs).^16,17^ HMOs are a diverse group of structurally complex glycans that constitute the third most abundant solid component of human milk.^18^ The concentration of HMOs varies among mothers and changes over time, depending on the stage of lactation.^19^ The capacity to metabolize HMOs by bacteria is highly species-specific, with certain species demonstrating superior proficiency in metabolizing these complex carbohydrates due to the presence of a large array of carbohydrate-active enzymes (CAZymes) and glycan transporters encoded in their genomes.^20–23^

With the rising concern over the early-life acquisition of antibiotic resistance, early interventions like breastfeeding could protect children against the intestinal colonization of antibiotic-resistant microorganisms (ARM).^24,25^ The species-specific capacity to utilize HMOs may play a crucial role in shaping the composition and functional dynamics of the infant gut microbiome and resistome. However, antibiotic treatment may disrupt the positive effect of HMO-degrading bacteria. The aim of this study was to explore the metabolism of HMOs by bifidobacteria and lactobacilli isolated from human milk and mother-infant paired fecal samples, as well as their antibiotic resistance profiles. Understanding these species-specific and sample-type-specific interactions will provide more evidence on the potential role of human milk in shaping the early-life resistome.

## Materials and methods

### Sample collection and ethics statement

A total of 39 maternal-infant fecal and mature human milk samples collected during the first month of life from the MAMI birth cohort^26^ were included in the study. Maternal-infant clinical records, including gestational age, neonate gender, birth weight, mode of delivery, type of lactation, and antibiotic exposure of the infant were collected (Supplementary Table S1). All infants were born term and were exclusively breastfed at the time of sample collection. All participants received oral and written information about the study, and written consent was obtained. The study was approved by the Ethical Committee of Hospital Clínico Universitario de Valencia and CSIC. The trial was registered on the ClinicalTrial.gov (number NCT03552939).

### Bacterial isolate collection

Bacterial isolations were performed in agar media including Difcto^TM^ Lactobacilli MRS Broth (Fisher Scientific, Hampton, NH, USA) supplemented with 0.05% cysteine and TOS Propionate Agar Base (Condalab, Madrid, Spain) supplemented with 0.005% mupirocin. For identification, morphologically unique colonies (1–7 maximum) were chosen from the agar media, re-streaked for isolation, grown in broth and viewed under a phase contrast microscope. Glycerol stocks were made from isolates and stored at −80 ºC for subsequent analyses. Colonies from the isolation step were further identified by sequencing of the 16S rRNA gene using the 27F-924 R primer pair (27F 5'-GAGTTTGATCMTGGCTCAG-3’ and 924 R 5'-CTTGTGCGGGYYCCCGTCAA-3’). The obtained sequence was searched on the BLAST database for the genus level taxonomic identification.

### Human milk oligosaccharide utilization assay of bacterial strains

#### Bacterial growth on a pool of human milk oligosaccharides

The ability of the isolated bacterial strains to utilize a pool of HMOs was assessed as previously described.^27^ Briefly, pooled HMOs (pHMOs) were extracted and purified following the method outlined by Jantscher-Krenn et al.^28^ Human milk samples, donated by healthy volunteers to a milk bank, were pooled and centrifuged to remove the lipid layer. Cold ethanol was added to precipitate proteins, which were then removed by centrifugation. Ethanol was eliminated via rotary evaporation. Lactose (Lac) and salts were removed using size exclusion and fast protein liquid chromatography. The resulting oligosaccharide mixture was lyophilized and stored at −20 °C until use.

Strains were cultured overnight in modified peptone-yeast-glucose broth (mPYG, Supplementary S1) under anaerobic conditions (5% H_2_, 10% CO_2_, balanced with N_2_) in a chamber (Whitley A45, Don Whitley Scientific, West Yorkshire, UK) to obtain the seed cultures for the assays. Assays were performed in diluted complex media, rather than minimal media, so that both positive and negative growth effects could be compared to a baseline level of growth. For anaerobic conditions, the media were degassed prior to inoculation.

Strains were grown under 4 different conditions: 1) mPYG dextrose-free medium, as the negative control (mPYG); 2) mPYG medium (mPYG_gluc); 3) mPYG dextrose-free medium supplemented with 15 g/L of pHMOs (pHMOs); 4) mPYG medium supplemented with 15 g/L of pHMOs (pHMOs_gluc). Each medium condition was inoculated with 5% (v/v) seed culture in biological triplicate in 96-well plates and overlaid with 50 μL sterile mineral oil to prevent evaporation. Plates were transferred into a BioTek Epoch 2 microplate spectrophotometer (Agilent, Santa Clara, CA, USA) for overnight growth measurements, which was placed under anaerobic conditions (5% H_2_, 10% CO_2_, balanced with N_2_). Plates were incubated for 24–48 h at 37 °C while optical density (OD_600_) readings were recorded every 30 min with 10s double orbital shaking immediately prior to each reading.

#### Individual HMOs and by-products utilization assays of bacterial strains

The growth of selected strains on individual HMOs or their degradation by-products was established as previously described,^29,30^ using the mPYG medium. For *Lactobacillaceae* strains, N-acetyllactosamine (LacNAc; Elicityl, Crolles, France) and lacto-N-neotetraose (LNnT; Glycom, Esbjerg, Denmark) were added individually to the mPYG dextrose-free medium at a concentration of 4 mm. For *Bifidobacterium* strains, lacto-N-tetraose (LNT; Glycom, Esbjerg, Denmark) and LNnT were added to the mPYG dextrose-free medium at a concentration of 2 mm. Glucose (Sigma-Aldrich, St. Louis, MO, USA), Lac (PanReac AppliChem, Barcelona, Spain) and galactose (PanReac AppliChem, Barcelona, Spain) were used as positive controls of growth, and mPYG dextrose-free medium was used as a negative control. Briefly, the strains were cultured overnight in mPYG medium and diluted to an OD_600_ of 0.1 in 200 μl of mPYG dextrose-free medium supplemented with each carbohydrate in 96-well plates. Cultures were grown at 37 ºC under the anaerobic conditions described above. Bacterial growth was determined by measuring the OD_600_ with Cerillo StratusTM Microplate Reader (Cerillo, Charlottesville, VI, USA) every 30 min with a constant shaking of 80 rpm for 48 h. At least two independent biological replicates and three technical replicates were performed for each growth assay.

#### HMO utilization by each bacterial strain

The utilization of HMOs was estimated by measuring the HMO profile of the bacterial growth supernatants. Bacterial cultures were centrifuged 10 min at 5,000 rpm and supernatants were collected and stored at −80 ºC. Each supernatant (20 μL) was spiked with maltose (20 mg/mL) as an internal standard at the beginning of sample preparation to correct for sample losses during sample processing and allow for absolute oligosaccharide quantification. Oligosaccharides were fluorescently labeled with 2‐aminobenzamide (2AB, Sigma) at 65 °C for 2 h. Labeled oligosaccharides were analyzed by high performance liquid chromatography (Dionex Ultimate 3000, Dionex, now Thermo Fisher) on an amide‐80 column (15 cm length, 2 mm inner diameter, 3 µm particle size; Tosoh Bioscience)] with a 50‐mmol L − 1 ammonium formate–acetonitrile buffer system. Separation was performed at 25°C and monitored with a fluorescence detector at 360 nm excitation and 425 nm emissions. HMO peaks were annotated based on standard retention times and quantified in reference to the internal standard.

The measured HMO structures included 2’-fucosyllactose (2′FL), 3-fucosyllactose (3FL), 3’-sialyllactose (3′SL), 6′-sialyllactose (6′SL), difucosyllactose (DFLac), LNT, LNnT, lacto-N-fucopentaose (LNFP) I, LNFP II, LNFP III, sialyl-lacto-N-tetraose b (LSTb), sialyl-lacto-N-tetraose c (LSTc), difucosyllacto-N-tetrose (DFLNT), disialyllacto-N-tetraose (DSLNT), lacto-N-hexaose (LNH), fucosyllacto-N-hexaose (FLNH), difucosyllacto-N-hexaose (DFLNH), fucodisialyllacto-N-hexaose (FDSLNH) and disialyllacto-N-hexaose (DSLNH).

### Antibiotic susceptibility testing on bacterial isolates

The resistance or susceptibility to antibiotics and the minimum inhibitory concentration (MIC) of bifidobacteria and lactobacilli isolates were determined following European Food Safety Authority (EFSA) guidelines^31^ and protocols described elsewhere.^32^ Following the definition used by EFSA, a bacterium was deemed susceptible when its growth was inhibited at a concentration of a specific antimicrobial equal to or lower than the established cutoff value (S ≤ x mg/l); and was deemed resistant when it was able to grow at a concentration of a specific antimicrobial compound higher than the established cutoff value (*R* > x mg/l). Hence, we performed the broth microdilution methodology to pre-screen which antibiotic resistances were exhibited by the isolates.

First, we prepared a battery of double-strength dilutions of the cutoff values of each antibiotic determined by the EFSA and distributed 50 µL in 96-well plates. Bacteria were grown in Difco^TM^ Latcobacilli MRS broth (Fisher Scientific, Hampton, NH, USA) supplemented with 0.05% cysteine, and 50 µL were inoculated into each well from a standardized suspension in the same broth medium for a final concentration of 3 × 10^4^ CFU/well. Plates were incubated for 48 h and the final OD_600_ was measured. Isolates showing resistance to any of the tested antibiotics were subject to obtaining MIC. For this, the same procedure as mentioned above was followed, but included a series of two-fold double-strength dilutions representing the final concentration range of dilutions established by EFSA.

### Whole genomic sequencing and data pre-processing

Bacterial isolates were grown overnight in a liquid culture and centrifuged for 6 min at 10,000 rpm to obtain the pellet for genomic DNA extraction. Briefly, each pellet was treated with lysozyme (20 ng/mL), liticase (500 U/mL) and mutanolysin (10 U/mL) for 40 min at 37 °C and cells were disrupted by bead-beating for 1 min at 6 m/s using 3-μm diameter glass and FastPrep 24-5 G Homogenizer (MP Biomedicals). Proteinase K (12 ng/mL) was added following an incubation of 20 min at 65 ºC and 15 min at room temperature. Next, MPC (Promega, Madison, WI, USA) was added to precipitate lipids and proteins, and the supernatant was taken into two consecutive centrifugations (15 min 13,000 rpm at 4 ºC) with 70% and 96% ethanol, respectively. RNA was removed by treating each washed pellet with RNAse A (5 mg/mL) for 1 h at 37 ºC. Finally, 3 M sodium acetate was added to precipitate the DNA, and another two consecutive centrifugations with 96% ethanol were performed. Finally, DNA was resuspended with 50 µL of MilliQ water for further purification and quantification. Purification of the DNA was performed using a DNA Purification Kit (Macherey-Nagel, Duren, Germany) according to manufacturer’s instructions. DNA concentration was measured using Qubit 2.0 Fluorometer (Life Technology, Carlsbad, CA, USA). Genomic DNA isolated from liquid culture was used for library preparation with Illumina DNA Prep and Tagmentation Kit (Illumina, San Diego, CA, USA), and was subjected to a cleaning step (0.6× Agencourt AMPure XP beads). Whole genome sequencing was performed on Illumina Sequencing Platforms (Novaseq6000 and Nextseq2000) at the Department of Cellular, Computational and Integrative Biology, University of Trento (Trento, Italy) and at the Quadram Institute Bioscience (Norwich, UK).

#### Genome annotation and phylogenetic analysis

Quality of the reads was assessed using FastQC (version 0.11.5).^33^ Specifically, reads were filtered for length, while those with a quality score less than Q30 were discarded using Trimmomatic (version 0.39).^34^ De-novo contigs were generated using SPAdes v4.0.0 (parameters: –careful -k 21,33,55,77,99,127).^35^ Assembly statistics computed using QUAST v5.1.0rc1.^36^ Genome completeness and contamination were estimated with CheckM v1.1.2.^37^ Mean coverage was computed using CMseq v1.0.4 (https://github.com/SegataLab/cmseq). Genomes with completeness >95% and contamination <1.5% were included in the downstream analyses. Taxonomic assignment was performed using GTDB-Tk v.2.4.0^38^ against bacterial and archaeal genomes based on the Genome Database Taxonomy (GTDB; Release 09–RS220 (24th April 2024)). Unless specified, all the computational tools used in this work were applied with default parameters. Average Nucleotide Identity (ANI) was calculated using FastANI (with default settings (K-mer size = 16, threads count for parallel execution = 1, fragment length default = 3,000).^39^ We analyzed multiple genomes using a genome list with command options of “—ql” and “–al.” For visualization, we used the “−matrix” command. For subsequent analysis, genomes with over 80% similarity were selected using FastANI. The assemblies and plasmid were annotated using Prokka (v.1.14.5).^40^ A phylogenetic tree was constructed using all species from *Bifidobacterium* and *Lactobacillaceae*, following the methodology with GToTree (v1.8.2),^41^ utilizing the protein sequences of 138 curated single-copy genes specific to *Actinomycetota* (i.e., *Bifidobacterium*), and 119 curated single-copy genes for *Bacillota* (i.e., *Lactobacillaceae*). Homologous sequences were aligned and concatenated using MUSCLE v3.8.1551.^42^ The resulting phylogenetic tree was constructed with FastTree v2.1.10.^43^ The tree was rooted using *Bifidobacterium animalis* subsp. *lactis* (*B. lactis*) for *Bifidobacterium* and *Lactobacillus paragasseri* for *Lactobacillaceae*. The iTOL web tool version 6^44^ was employed for the tree visualization and annotation.

#### Genomics-based reconstruction of HMO metabolism

We used a subsystems-based approach implemented in the SEED platform^45^ for the reconstruction of HMO utilization pathways across the set of 39 *Bifidobacterium* and 14 *Lactobacillaceae* genomes. The genomes were annotated via RAST^46^ and uploaded to mcSEED (microbial community SEED), a clone of the SEED annotation environment. Homology and genomic context-based methods in mcSEED were used to analyze the representation of groups of orthologous genes implementing a curated set of 67 and 57 functional roles in bifidobacteria and lactobacilli, respectively, that contribute to HMO metabolism. These functional roles included components of glycan transporters, extracellular and cytoplasmic glycoside hydrolases (GHs), monosaccharide catabolic enzymes, and transcriptional regulators.^23,47^ The genomic distribution of functional roles was used to reconstruct glycan utilization pathways in two metabolic subsystems containing the genomes of bifidobacteria and lactobacilli isolates, respectively. For bifidobacteria, we reconstructed pathways for Lac, lacto-N-biose (LNB), LNT, LNnT, 2’FL, 3FL, DFLac, LNFP I, 3’SL, 6’SL, and lcHMO catabolism (Supplementary Figure S2 and Supplementary Table S2). The lcHMOs are HMOs with a degree of polymerization ≥5 (e.g., LNFP II/III, LNDFH I/II) for which separate utilization pathways could not be unequivocally predicted due to the insufficient information on the substrate specificity of corresponding HMO transporters. For lactobacilli, pathways for individual HMO (LNT and LNnT), and the products of HMO degradation (Lac, LacNAc, LNB, and lacto-N-triose [LNTriose], L-fucose [Fuc], N-acetylneuraminic acid [Neu5Ac], N-acetyl-D-glucosamine [GlcNAc], D-galactose [Gal], and D-glucose [Glc]) were reconstructed (Figure 4(b) and Supplementary Table S3).

The reconstructed pathways contained alternative biochemical routes implemented by different subsets of catabolic enzymes and diverse glycan transporters corresponding to different ecological strategies of HMO metabolism (extracellular vs. intracellular). To enable comparison across different strains, the detailed pathway variants were simplified to binary utilization phenotypes (1 and 0) corresponding to the predicted ability to metabolize a specific HMO species or its degradation by-products.

#### Identification of antibiotic resistance genes, virulence factors and plasmids

The antimicrobial resistance genes (ARGs) were detected from the assembly output of Resistance Gene Identifier (RGI) v6.0.3 with the CARD database v3.3.0,^48^ and Strict significance based on CARD curated bitscore cutoffs. The virulence factors were identified by ABRicate v1.0.1 (https://github.com/tseemann/abricate.) using the Virulence Factor Database (VFDB)^49^ with a cutoff coverage of 80 and 70% identification. Plasmids were detected by the Platon v1.7,^50^ which is based on a taxon-independent approach to extract both plasmid and genomic sequences. The pathogenicity toward humans was predicted by PathogenFinder v1.1 (https://cge.food.dtu.dk/services/PathogenFinder/).

### Statistical analysis

All statistical analyses were performed using R version 3.6.0, and Figures were created with the *ggplot2* R package.^51^ For all methods, p-values were adjusted for multiple comparisons using the Benjamini-Hochberg (BH) method.^52^ Normality of the data was evaluated using Shapiro-Wilk tests. Categorical variables are expressed as positive cases-prevalence and (percentage, %). Normally distributed data are presented as mean ± standard deviation (SD) and non-normal data as median and interquartile range [IQR]. Pearson’s Chi-square test or Fisher’s exact test was used for categorical variables, and the Mann-Whitney U test was applied to continuous variables, to assess statistical significance.

#### Growth curve analysis

Growth curves were analyzed using the R package *gcplyr*^53^ which carries out model-free and non-parametric analyses to directly quantify attributes of growth dynamics. The underlying OD_600_ of the base medium was subtracted from each condition at each timepoint (blank subtraction). The area under the curve (AUC), the maximum growth rate (r) and carrying capacity (k) were calculated to study the impact of HMOs on the growth properties of the strains.

Log fold changes in growth curve metrics were calculated by dividing the values of the growth curve metric from condition 1 by condition 2 and applying logarithmic transformation. Growth curve metrics between the different conditions of each strain were compared by Mann-Whitney U tests with p-values adjusted using the BH method.

#### HMO utilization analysis

HMO utilization at 24–48 h was calculated in reference to the initial HMO concentrations in the medium at the beginning of each experiment (*t* = 0) and represented as the percentage of HMOs remaining in the medium. The *ComplexHeatmap* R package^54^ was used to draw the HMO utilization profile of each group of species.

#### Resistance and HMO consumption profiles association analyses

Bray-Curtis distance-based Multivariate Redundancy Analysis (db-RDA) was conducted to test the correlation between HMO consumption capacity and antibiotic resistance profiles, by terms of MIC values. Both datasets where log transformed and the mean of overall HMO consumption and MIC values of each species were used for db-RDA. Antibiotic susceptibility was indicated as a MIC value of 0. The ‘adonis2’ function from *vegan* R package^55^ was used to perform permutations to evaluate overall differences in HMO consumption between species and to assess the association with the resistance profile (taken as the variables). Mann-Whitney U tests with p-values corrected using the BH method were performed to study the differences between the log transformed mean HMO consumption values and the log transformed mean MIC values of each species. The significance value was determined based on 999 permutations.

## Results

### *Targeted isolation and genome sequencing of* Bifidobacteriaceae *and* Lactobacillaceae *strains*

**Figure 1.**
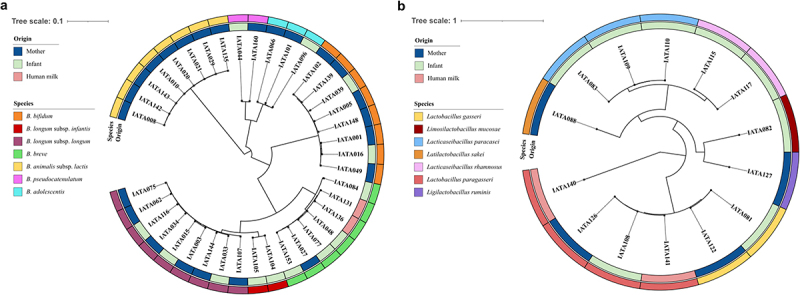
Phylogenetic trees of *Bifidobacteriaceae* (a) and *Lactobacillaceae* (b) isolates from mother-infant pairs.

Isolates from maternal-infant paired fecal and human milk samples identified as *Bifidobacterium* (*N* = 39) or *Lactobacillaceae* (*N* = 14) with a mean genome coverage of 95.59% were included in this study (Figure 1). The median genome size was 2.32 Mb with an average of 44.24 contigs per genome and the GC content of the genomes varied in the range between 34.74% and 62.90%, with a median of 59.63% (Supplementary Table S4).

In summary, 22 (39.28%) unique isolates were obtained from infant fecal samples (*N* = 15), 30 (53.71%) from maternal fecal samples (*N* = 23), and 4 (77.14%) from human milk (*N* = 3) (Supplementary Table S1). The 39 *Bifidobacterium* strains belonged to 6 species: 3 *B. adolescentis*, 8 *B. lactis*, 8 *B. bifidum*, 7 *B. breve*, 2 *B. pseudocatenulatum*, 2 *B. infanti*s and 9 *B. longum* subsp. *longum* (*B. longum*). The 14 *Lactobacillaceae* strains represented 7 species: 4 *Lacticaseibacillus paracasei*, 2 *Lacticaseibacillus rhamnosus*, 2 *Lactobacillus gasseri*, 4 *Lactobacillus paragasseri*, 1 *Lactilactobacillus sakei*, 1 *Limosilactobacillus mucosae*, and 1 *Ligilactobacillus rumnis*.

### Bacterial growth on pHMOs

**Figure 2.**
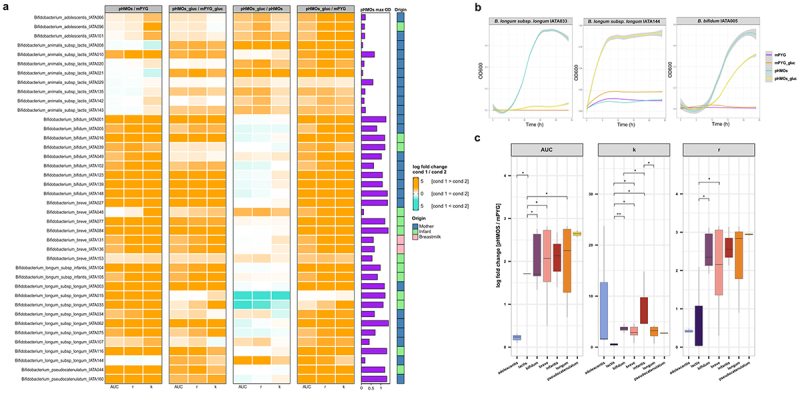
Growth of *Bifidobacterium* isolates on human milk oligosaccharides. (a) Growth of the 39 *Bifidobacterium* strains isolated from human milk and mother-infant fecal samples in diluted media supplemented with 4 carbon sources: pHMOs (pHMOs), pHMOs and glucose (pHMOs_gluc), glucose (mPYG_gluc), and no carbon source (mPYG). Log fold changes of area under the curve (AUC), carrying capacity (k), and growth rate (r) between the two conditions tested (condition 1/condition 2) are represented. A gradient-colored in orange represents a higher growth curve metric of condition 1 relative to condition 2, whereas a gradient-colored in blue represents a higher metric of condition 2 relative to condition 1. Horizontal bars in purple represent the highest OD reached in the pHMOs condition. The origin of each isolate is indicated as a column annotation (from mother feces in blue, from infant’s feces in green, and from human milk in pink). Supplementary Table S5 shows significant comparisons for each growth metric between treatments for each *Bifidobacterium* strain. (b) Growth curves for *B. longum* IATA033, IATA144, and *B. bifidum* IATA005 under the four growth conditions. (c) Mean log fold change between the pHMOs and mPYG conditions of the *Bifidobacterium* strains grouped by species. *p < 0.05, **p < 0.01.

The growth of all *Bifidobacterium* isolates in the presence of glucose and/or pHMOs was assessed (Figure 2(a)). All *B. bifidum* strains were able to grow with pHMOs as the sole carbon source, as indicated by a positive log fold change of pHMOs/mPYG. For most of the strains, pHMOs were a more favorable energy source as strains reached a slightly higher AUC and r compared to pHMOs_gluc treatment (Figure 2(b)). Most isolates of *B. breve* and *B. infantis* displayed similar growth properties as the *B. bifidum* isolates. In addition, the growth of the two *B. pseudocatenulatum* strains tested was enhanced in the presence of pHMOs.

*B. longum* isolates showed strain-level heterogeneity in their growth properties under the various conditions tested. For *B. longum* strains IATA015, IATA033, IATA062 and IATA075, pHMOs resulted in a higher k compared to mPYG_gluc (Figure 2(a,b)). In contrast, *B. longum* IATA144 did not grow in the presence of only pHMOs (Figure 2(b)). The remaining *B. longum* strains grew better in the presence of glucose compared to pHMOs despite an ability to utilize pHMOs as a sole carbon source. Interestingly, all *B. adolescentis* strains and most *B. lactis* strains, including IATA135, IATA143, IATA021, and IATA008, tested were incapable of using pHMOs as the sole carbon source but displayed enhanced growth in the presence of both pHMOs and glucose compared to glucose only. On the other hand, the growth of *B. lactis* IATA020, IATA029 and IATA142 was not affected by pHMOs. Only *B. lactis* IATA010 displayed an ability to metabolize pHMOs as the sole carbon source. Overall, *B. lactis* displayed the lowest capacity to grow on pHMOs alone, as strains displayed a lower log fold change of the AUC, r and k compared to other species tested (Kruskal–Wallis test, *p* < 0.05) (Figure 2(c)). The ability of *Bifidobacterium* strains to metabolize HMOs was influenced by their isolation source. Strains isolated from infant stool produced a higher k (log fold change 5.18 ± 2.87) than those isolated from mother’s stool (log fold change: infants = 5.18 ± 2.87, mothers 2.17 ± 1.71, Wilcoxon test, *p* = 0.04).

Among the *Lactobacillaceae* strains tested, growth with pHMOs as the sole carbon source was poor (OD_600_ < 0.3). However, the strains displayed enhanced growth when both pHMOs and glucose were added to the medium, surpassing the growth when only glucose was present (Supplementary Figure S1). There were no significant differences in the activities of the *Lactobacillaceae* strains, nor did their behavior vary statistically based on their isolation source.

#### Genotype-to-phenotype predictions of HMO utilization

**Figure 3.**
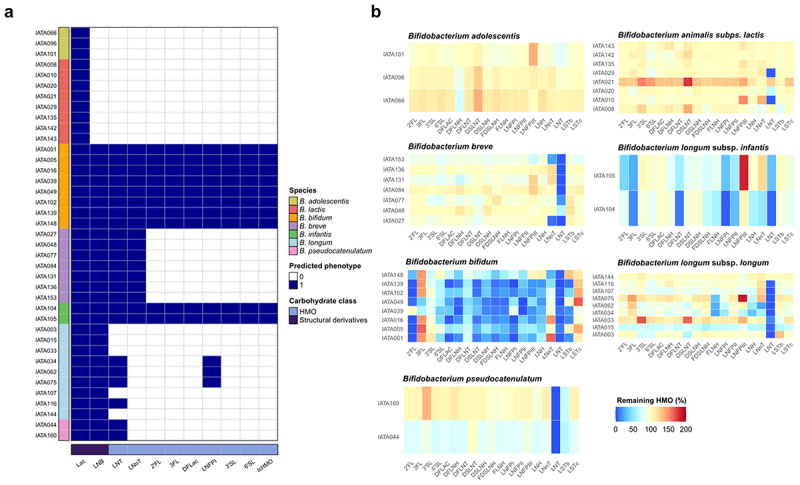
Genotype-to-phenotype associations of HMO metabolism in *Bifidobacterium* isolates. (a) Representation of reconstructed utilization pathways for HMOs and their structural components in bifidobacterial strains. The presence of a specific pathway (blue) corresponds to a predicted ability to metabolize the corresponding glycan. (b) Remaining HMO (%) in the culture medium supernatant after 48 h of growth of each *Bifidobacterium* isolate.

We analyzed the representation of reconstructed HMO utilization pathways encoded in the genomes of *Bifidobacterium* strains and associated them with their HMO utilization profiles, expressed as the percent of remaining HMOs in the culture supernatant after 48 h of growth (Figure 3 and Supplementary Figure S2). A detailed description of genotype–phenotype comparisons can be found in Supplementary Note S2. Overall, the *in silico* predictions were in good agreement with *in vitro* HMO utilization profiles for most strains; however, several discrepancies were observed.

*B. bifidum* strains degraded most measured HMO structures, consistent with the presence of multiple HMO-acting extracellular GHs encoded in the genomes of these strains (Supplementary Table S2). However, some strain-level variability of HMO utilization was noted. For example, *B. bifidum* IATA039 could not degrade 2’FL, likely due to a 5’-end truncation of the gene encoding the alpha-1,2-fucosidase (BbAfcA; GH95) required for 2’FL metabolism^56^ (Supplementary Figure S3). We also observed an interplay between concentrations of 3FL and DFLac in the supernatants of all strains except *B. bifidum* IATA049 and IATA039 (Figure 3(b)). The increased 3FL concentration could be explained by the unequal (separated in time) removal of fucose residues from DFLac by the alpha-fucosidases of *B. bifidum*. The concentration of LNnT in the supernatants of *B. bifidum* strains varied considerably, decreasing in some strains (IATA049, IATA102, IATA139, IATA148) and increasing in others (IATA001, IATA016). Since LNnT is an intermediate product of the degradation of multiple more complex HMOs (e.g., LNFP III), the observed results may reflect strain-level variability in the kinetics of long-chain HMO degradation. *B. bifidum* strains IATA102, IATA005, IATA001, and IATA049 were able to grow in the medium supplemented with LNnT as a sole carbon source, confirming their capacity to utilize this HMO (Supplementary Figure S4a).

*B. infantis* strains efficiently utilized multiple HMO species, including 2’FL, 3FL, LNFP I, LNT, LNFP II, and DFLNT, consistent with the *in silico* predictions (Figure 3(a)). *B. infantis* IATA105, however, did not utilize LNnT, potentially due to mutations in genes encoding the respective utilization pathway. *B. longum* strains displayed varying capacities to utilize HMOs. *B. longum* strains IATA034, IATA062, and IATA075 degraded LNT, LNFP I, LNH, and FLNH via a mechanism involving an extracellular lacto-N-biosidase (LnbX; GH136)^57^ (Supplementary Figure S2). Other *B. longum* strains utilized only LNT, likely via an intracellular mechanism, although the variant of the LNT transporter (GltABC) found in most of these strains was previously shown to have low affinity to LNT.^58^

All *B. pseudocatenulatum* strains were predicted to utilize LNT, which was confirmed by their ability to consume over 95% of the LNT present in the pHMO mixture (Figure 3(a,b)). In agreement with *in silico* predictions, *B. breve* IATA027 and IATA153 efficiently consumed both LNT (>90%) and LNnT (>75%) (Figure 3(b)). The remaining *B. breve* strains depleted only LNT. Consistent with glycoprofiling data, *B. breve* IATA048 and IATA131 grew in a medium supplemented with LNT but not LNnT (Figure 3(c)). The discrepancy between genomic predictions and experimental observations of LNnT utilization could be attributed to mutations in the genes involved in the metabolism of this oligosaccharide. For example, in *B. breve* IATA131, the *nahS* gene was disrupted by a premature stop codon, suggesting that the encoded substrate-binding component of the LNnT-specific ABC transporter might be nonfunctional (Supplementary Figure S3).

*B. adolescentis* and *B. lactis* strains were predicted to lack the capability to utilize any HMOs due to the absence of required transport systems and GHs (Figure 3(a) and Supplementary Table S2). Glycoprofiling results supported this prediction for most strains. Unexpectedly, *B. lactis* strains IATA010, IATA020, and IATA029 depleted LNT from the pHMO mixture and grew in a medium where LNT served as the sole carbon source (Supplementary Figure S4a).

**Figure 4.**
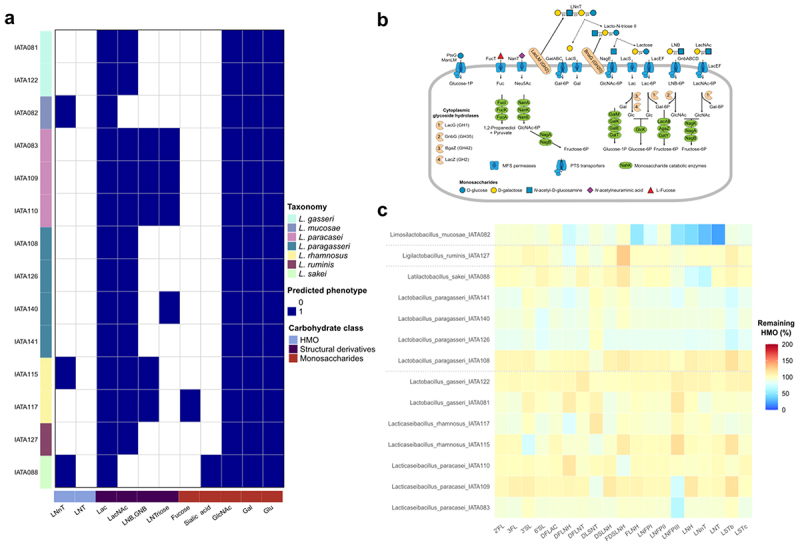
Genotype-to-phenotype associations of HMO metabolism in *Lactobacillaceae* isolates. (a) Representation of reconstructed utilization pathways for HMOs and their structural components in *Lactobacillaceae* isolates. The presence of a specific pathway (blue) corresponds to a predicted ability to metabolize the corresponding glycan. (b) Schematic representation of reconstructed HMO and their degradation by-products utilization pathways. (c). Remaining HMOs (%) in the culture medium supernatant after 24 h of growth of each lactobacilli isolate.

We also analyzed the representation of HMO utilization pathways in 14 *Lactobacillaceae* and compared it with HMO glycoprofiling data (Figure 4 and Supplementary Table S3). Unlike the bifidobacteria tested, most lactobacilli except *L. mucosae* IATA082, *L. rhamnosus* IATA115, and *L. sakei* IATA088 were predicted not to utilize any HMO structures. Despite lacking complete HMO utilization pathways, all analyzed strains were predicted to metabolize mono- and disaccharide products of HMO degradation, namely, lactose, N-acetylglucosamine, galactose, and glucose, and some strains were predicted to also utilize N-acetyllactosamine, lacto-N-biose, and lacto-N-triose II (Figure 4(a,b)).

*L. mucosae* IATA082, *L. rhamnosus* IATA115, and *L. sakei* IATA088 were predicted to partially degrade LNnT via a mechanism involving an extracellular beta-galactosidase (Supplementary Table S3). Consistent with this prediction, *L. mucosae* IATA082 and *L. sakei* IATA088 depleted LNnT from the pHMOs mixture and grew in the medium with LNnT as the sole carbon source (Figure 4(c) and Supplementary Figure S4b). Notably, these were the only strains lacking the N-acetyllactosamine utilization pathway and failed to grow when N-acetyllactosamine was the only carbon source in the medium (Supplementary Figure S4b). *L. rhamnosus* IATA115, however, did not degrade LNnT present in the pHMOs mixture. Other discrepancies between genomic predictions and experimental data include the unexpected partial degradation of LNFP III by *L. paracasei* strains IATA083 and IATA109 (Figure 4(c)). Additionally, *L. mucosae* IATA082 depleted LNT, LNH as well as fucosylated HMOs (LNFP III, FLNH, DFLNH) despite lacking genes encoding alpha-fucosidases (Figure 4(c) and Supplementary Table S3).

### Genotype-to-phenotype predictions of antibiotic resistance

**Figure 5.**
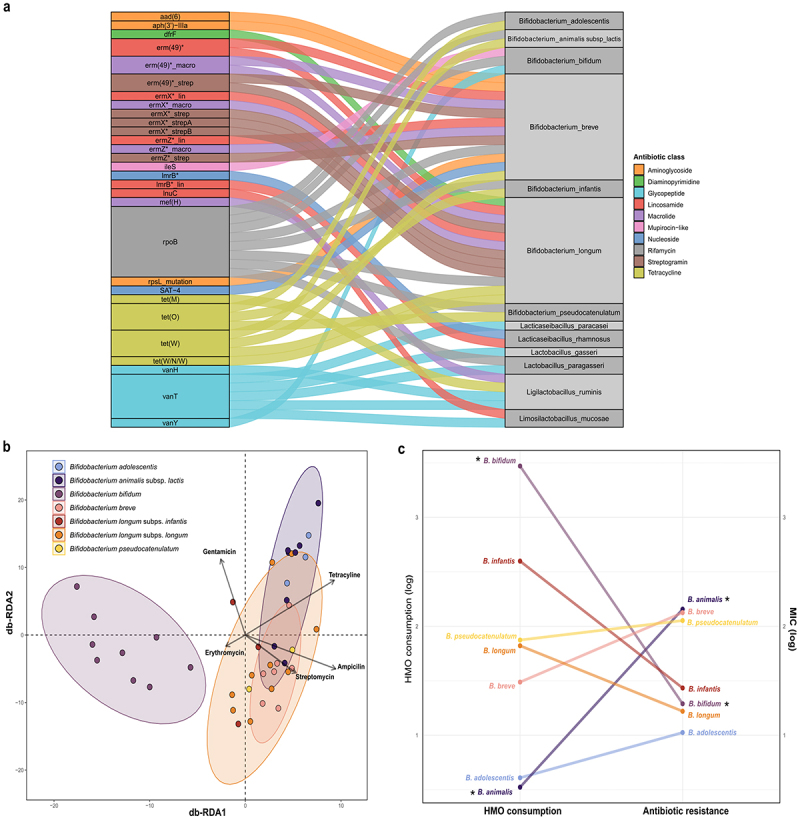
ARGs present in the genome of the *Bifidobacteriaceae* and *Lactobacillaceae* isolates and their association with HMO metabolism. (a) Summary of the ARGs detected in each of the bacterial genomes, coloured by class of antibiotic resistance. An asterisk (*) next to the gene name indicates that it encodes for multiple resistance factors. Bacterial genomes are grouped by species. (b) Distance-based redundant discriminant analysis (db-RDA) biplot depicting the relationship between the HMO degradation profile and antibiotic resistance of each strain, coloured by species of *Bifidobacterium*. in db-RDA, angles between the arrows represent correlations; acute angles represent positive correlations and obtuse angles represent negative correlations. Pairwise PERMANOVA (adonis-pairwise) was performed to evaluate overall differences in HMO consumption between species and to assess the association with the resistance profile (taken as the variables). Mann-Whitney U tests with p-values corrected using the BH method was performed to study the differences between the log transformed mean HMO consumption values and the log transformed mean MIC values of each species. (c) Differences in HMO consumption (log of remaining HMOs) and antibiotic resistances (log MIC values) of *Bifidobacterium* species. Susceptibility to an antibiotic was considered as a MIC value of 0. An asterisk (*) indicates differences that were significant (*p* < 0.05).

RGI analysis revealed 26 ARGs in bifidobacteria and 8 ARGs in the lactobacilli isolates, with perfect and strict hits (Supplementary Figure S5). These genes were predicted to have >80% sequence identity to well-characterized ARGs in the CARD database. The antibiotic resistance phenotypes (Supplementary Tables S7, S8 and Supplementary Note S3) and genotypes (Figure 5(a)) of the *Bifidobacterium* and *Lactobacillaceae* strains were compared.

Phenotypically, all *Bifidobacterium* strains exhibited susceptibility to vancomycin and in concordance, no glycopeptide resistance genes were found in any of the genomes. On the contrary, all bifidobacteria were resistant to erythromycin, although *erm(49)* and *ermX* macrolide-streptogramin resistance genes were only identified in *B. breve* and *B. longum* strains. Although no beta-lactam resistances were identified at the genome level, 89.7% of the bifidobacteria were resistant to ampicillin.

The *tetW* gene, which confers resistance to tetracycline, was identified in the genomes of strains of *B. lactis, B. longum* and *B. breve*, all of which phenotypically exhibited resistance to tetracycline. In contrast, all *B. bifidum* and *B. infantis* strains, as well as 77.7% of *B. longum* strains, were susceptible to tetracycline. Interestingly, all *B. infantis* strains carried a *tetO* resistance gene despite displaying a susceptibility to tetracycline.

Resistance to aminoglycosides was tested experimentally with gentamicin and streptomycin. Only three *B. breve* strains carried two aminoglycoside resistance genes in their genomes (*aad6* and *aph(3’)-Illa*). However, only *B. breve* IATA153 exhibited resistance to both gentamicin and streptomycin, while *B. breve* IATA048 and IATA077 were susceptible to gentamicin. Although no aminoglycoside resistance genes were found in their genomes, *B. bifidum*, *B. infantis*, and *B. longum* strains were generally resistant to gentamicin but susceptible to streptomycin.

All *Lactobacillaceae* strains were susceptible to chloramphenicol, while only *L.sakei, L. mucosae*, and *L. ruminis* strains were resistant to tetracyclines. Indeed, the *tetM* gene was identified in the genomes of *L. ruminis* isolates. *L. rhamnosus* strains showed lower levels of resistance. Namely, *L. rhamnosus* IATA117 was susceptible to all antibiotics tested and *L. rhamnosus* IATA116 was resistant to only erythromycin and ampicillin.

Although not tested *in vitro*, other resistances were detected in the genomes of the *Bifidobacterium* and *Lactobacillaceae* strains. These included resistance to lincosamides, mainly present in *B. breve*, *B. longum*, *L. rhamnosus* and *L. mucosae* strains. All *Bifidobacterium* strains contained *rpoB* mutants that confer resistance to rifampicin. Mupirocin-like resistance was identified in 9 bifidobacterial strains. Overall, these results emphasize the importance of combining *in silico* and *in vitro* analyses to confirm that the detected ARGs are phenotypically expressed.

Genomic analyses also allowed the detection of lateral gene transfer (LGT) events in each strain, as well as the presence of plasmids (Supplementary Table S9). All *B. infantis* strains and *B. longum* IATA116 possessed the potential ability to exchange genes with species of *Clostridium*, *Eubacterium*, *Blautia* or *Coprococcus*. Similarly, *B. bifidum* IATA005 and IATA049 could potentially transfer genes to members of *Propionibacterium* and *Coprococcus*. On the other hand, the potential for LGT was detected in 6 of the 14 *Lactobacillaceae* isolates included in this study. *L. mucosae* IATA082 displayed the potential to transfer genes to *L. fermentum* species. Moreover, the other lactobacilli strains displaying LGT capacity could both acquire genes from or transfer genes to *Enterococcus* spp. Finally, the plasmids detected in the bacterial isolates were primarily mobilization or replication plasmids, although some strains carried conjugation plasmids.

### Associations between antibiotic resistance and HMO metabolism

We performed db-RDA to examine the potential association between HMO metabolism capabilities and the antibiotic resistance profiles of the bacterial isolates (Figure 5(b)). The analysis demonstrated that bifidobacterial strains of the same species clustered together according to their HMO metabolism capacity. *B. bifidum* strains formed a distinct cluster in the db-RDA ordination, supported by significant separation from *B. lactis* (R^2^ = 0.602, *p* = 0.021), *B. breve* (R^2^ = 0.561, *p* = 0.042), and *B. longum* (R^2^ = 0.539, *p* = 0.021), as determined by PERMANOVA. Moreover, resistance to ampicillin (R^2^ = 0.196, *p* = 0.001) and tetracycline (R^2^ = 0.883, *p* = 0.016) were not associated with the *B. bifidum* cluster.

In fact, the contrast between the high capacity to consume HMOs and the low antibiotic resistance phenotype of *B. bifidum* was highly significant (Mann-Whitney U, *p* = 0.003) (Figure 5(c)). A similar trend was observed in *B. longum* and *B. infantis*, though the differences were not statistically significant. In contrast, other *Bifidobacterium* species, such as *B. lactis*, had a lower capacity for HMO utilization but a higher resistance to antibiotics (Wilcoxon test, *p* = 0.0003).

The db-RDA analysis for *Lactobacillaceae* isolates did not yield conclusive results, primarily due to their limited ability to utilize HMOs (Supplementary Figure S6). *L. mucosae* IATA082 was the only strain capable of degrading HMOs and, accordingly, clustered separately from the other *Lactobacillaceae* strains, although this separation was not statistically significant. Overall, the *Lactobacillaceae* strains lacked both the ability to metabolize HMOs and resistance to the tested antibiotics, and no association between these two independent traits was observed.

## Discussion

With growing concern over the early-life acquisition of antibiotic resistance, interventions such as breastfeeding may offer protection by limiting intestinal colonization with antibiotic-resistant microorganisms.^12^ One proposed mechanism involves HMOs, which selectively nourish beneficial bacteria in the infant gut. Species-specific HMO utilization may significantly influence the composition and function of the infant gut microbiome and indirectly affect early-life acquisition of antibiotic resistance. However, external factors like antibiotic treatment can disrupt this process by hindering the establishment of HMO-degrading bacteria, thereby reducing their positive effects. This disruption may favor the growth of pathogenic and antibiotic-resistant microorganisms.^59^ Understanding the strain-level capabilities for HMO metabolism and the impact of antibiotics on early-life colonizers is therefore critical for evaluating the long-term consequences for the infant resistome.

This study aimed to enhance our understanding of strain-specific HMO metabolism among early-life gut colonizers and to investigate how antibiotic exposure may influence these microbes. We found that HMO utilization by bifidobacteria is highly species-specific. *B. bifidum* and *B. infantis* exhibited the most extensive capacity to degrade HMOs, consistent with their genomic profiles, and are known early colonizers of the breastfed infant gut. In contrast, *B. longum* and *B. breve* showed more limited HMO-degrading capabilities, although some *B. longum* strains possessed genes encoding extracellular lacto-N-biosidase, supporting a stronger metabolic potential. These findings are consistent with previous studies that have documented interspecies variation in HMO utilization and validated corresponding metabolic pathways at the genomic level.^17,23,60,61^ On the other hand, *B. pseudocatenulatum, B. adolescentis* and *B. lactis*, species commonly associated with the adult gut, did not efficiently degrade HMOs and lacked the necessary genetic machinery for their metabolism, in concordance with previous literature.^62^

Interestingly, some *Bifidobacterium* strains exhibited enhanced growth and more efficient HMO consumption in the presence of HMOs compared to glucose or HMOs and glucose combined. This highlights their specific and unique capacity to metabolize HMOs efficiently and suggests a potential role for glucose metabolism in regulating HMO-degrading pathways. However, further experiments are needed to validate these observations.

Regarding antibiotic resistance, *B. bifidum* strains showed the lowest resistance levels, whereas *B. lactis* displayed resistance to multiple antibiotics. Notably, *B. bifidum* emerged as the species with the highest HMO degradation capacity and the lowest antibiotic resistance, emphasizing its potential significance in early-life gut colonization. Together, these findings underscore the pivotal role of breastfeeding in shaping the infant gut microbiome by promoting the growth of HMO-degrading bacteria, particularly bifidobacteria. At the same time, our results highlight the potential disruptive effects of antibiotic exposure in early life, which could interfere with the establishment of key microbial species critical for infant health.

In the case of *Lactobacillaceae*, it has been previously reported that their ability to utilize HMOs is not as widespread as *Bifidobacterium* members.^17,20,21^ In line with this, we identified fewer HMO-active GHs in the genomes of the *Lactobacillaceae* strains compared to the *Bifidobacterium* strains. Many *Lactobacillaceae* members are capable of metabolizing HMO-derived components, such as glucose, Lac, LacNAc, LNB and GlcNAc, to support their growth, classifying them as “secondary degraders”.^63,64^ Consistent with these reports, we identified the relevant metabolic pathways in *L. rhamnosus* and *L. paracasei*. Furthermore, in both our *in vitro* and *in silico* analyses, *L. mucosae* emerged as one of the most versatile carbohydrate-degrading *Lactobacillaceae* species, followed by *L. sakei*, with no previous literature documenting these findings.

As observed in this study, HMO degradation profiles vary significantly among species and their coexistence in the infant gut microbiota may reflect complex metabolic interactions.^65–67^ Certain species possess enzymes that cleave more complex HMOs to produce by-products that can be degraded by other taxa, thus acting as a driver of community structure through the provision of substrates for others to consume.^68^ For example, *Lactobacillaceae* members may depend on the presence of bifidobacteria or other gut microbes to liberate di- and monosaccharide components from HMOs to support their growth.^69^ Although the present study focused on HMO metabolism in monocultures, future studies involving co-cultures or complex fecal microbial communities could provide deeper insights into these interspecies metabolic interactions and their role in early-life gut microbiome development.

We found that both bifidobacteria and lactobacilli strains harbored ARGs and exhibited resistance *in vitro*, despite reports that they carry fewer ARGs than other genera and are less prone to horizontally transfer of ARGs.^70–72^ In some cases, the resistance may be the result of punctual mutations in specific genes.^32,73^ *B. bifidum* and *B. infantis* were more susceptible to antibiotics than *B. lactis* and *B. adolescentis*. *L. sakei, L. mucosae*, and *L. ruminis* strains were resistant to tetracyclines, though the majority of the identified genotypic resistances were related to glycopeptides. Given the widespread acceptance of lactic acid bacteria as probiotics with established benefits for infant gut health, the ability of some strains to survive antibiotic exposure could potentially be advantageous. For example, *B. pseudocatenulatum* strains have previously been noted for their antibiotic survivability in this context.^74^ While some bifidobacterial strains exhibited intrinsic resistance to antibiotics, generally considered low-risk for horizontal transfer,^73^ our analysis of LGT and plasmid content revealed the potential cross-species exchange of ARGs between bifidobacteria, lactobacilli, and other gut microbes.

Discrepancies between the *in silico* and *in vitro* antibiotic resistance profiles also emerged in our findings. Discrepancies may arise from regulatory mechanisms, mutations that disrupt the potential function, or new and/or evolving resistance mechanisms not captured in ARG reference databases. Moreover, a strain may be resistant to an antibiotic although no related ARGs are identified. For example, we found that *B. lactis* strains were the most resistant to antibiotics despite their genomes containing only two known ARGs. This highlights the critical need to consistently corroborate genomic predictions with phenotypic results.

Our findings revealed an inverse relationship between HMO degradation capabilities and antibiotic resistance. Strains such as *B. bifidum, B. infantis*, and *B. longum*, frequently isolated from infant sources, efficiently degrade a variety of HMOs yet remain highly susceptible to antibiotics. Conversely, *B. lacti*s and *B. adolescentis* exhibit greater antibiotic resistance but a reduced ability to utilize HMOs. Only one previous study has linked the development of the infant resistome to microbial carbohydrate metabolism during early life.^25^ That study, using metagenomic functional profiling and metagenome-assembled genome analyses, demonstrated that shifts in microbial carbohydrate metabolism correspond to the temporal development of the gut resistome. Our findings align with these previous reports and are further supported by our *in* *vitro* observations. These results underscore the crucial role of breastfeeding in shaping the infant gut microbiome by fostering the growth of HMO-degrading bacteria, particularly bifidobacteria. However, early-life antibiotic exposure may disrupt this process by depleting these beneficial bacteria, thereby favoring antibiotic-resistant strains. Since early-life colonizers are highly susceptible to antibiotics, their suppression could impair microbial cross-talk, such as interactions between bifidobacteria and lactobacilli, ultimately compromising the protective effects of breastfeeding and HMOs in mitigating antibiotic resistance. Given their ability to selectively promote beneficial bacteria, HMOs hold promise as a potential therapeutic to support microbiome resilience and counteract the adverse effects of early-life antibiotic exposure.

In conclusion, our findings demonstrate that temporal variation in *Bifidobacterium* and *Lactobacillaceae* species in the infant gut is influenced not only by their specific ability to utilize HMOs but also by their antibiotic resistance profiles. We have highlighted that early-life antibiotic exposure can disrupt colonization by beneficial HMO-degrading bacteria, impeding their role in supporting a balanced gut microbiome, while, in turn, promoting the growth of antibiotic-resistant bacteria. Future research should explore the transmission dynamics of these species and their role as ARG reservoirs in complex environments to elucidate the development and trajectory of the infant resistome. This includes investigating the role of breastfeeding, human milk composition, and the impact of antibiotic treatment, with the ultimate goal of improving the monitoring and management of antibiotic prescriptions.

### Strengths and limitations

This study employed a combination of methods to model the prediction of antibiotic resistance and the degradation of HMOs, focusing on bifidobacteria and lactobacilli within the maternal-infant microbiotas. By identifying ARGs, the study assessed the impact of antibiotic use on neonatal gut colonization, laying groundwork for strategies to maintain beneficial microbiota and curb early-life antibiotic resistance spread. Culturing bacteria from mother-infant pairs and examining glucose’s effect on HMO degradation provided insights into microbial metabolism. However, the study faced limitations including a small sample size, reducing the generalizability of the findings. Its cross-sectional, region-specific design restricted exploration into microevolution and comparisons across different geographic populations. Additionally, differences in diet between breastfed infants and adults complicated comparisons of HMO degradation and antibiotic resistance among strains. Future research should focus on expanding the collection and analysis of beneficial strains, incorporating broader genomic data, and conducting longitudinal studies to better understand microbial adaptation and the persistence of beneficial strains in the gut over time.

## Supplementary Material

Supplemental Material

## Data Availability

Genome sequences of all isolates have been deposited in the National Center for Biotechnology Information (NCBI) under accession PRJNA1190840.

## Abbreviations

HMO: Human Milk Oligosaccharides
ARG: Antibiotic Resistance Gene:
ARM: Antibiotic Resistance Microorganism
2’FL: 2′-fucosyllactose
3FL: 3-fucosyllactose
3’SL: 3′-sialyllactose
6’SL: 6′-sialyllactose
DFLNH: Difucosyllacto-N-hexaose
DFLNT: Difucosyllacto-N-tetraose
DFLac: Difucosyllactose
DSLNH: Disialyllacto-N-hexaose
DSLNT: Disialyllacto-N-tetraose
FDSLNH: Fucodisialyllacto-N-hexaose
FLNH: Fucosyllacto-N-hexaose
LNFP I: Lacto-N-fucopentaose I
LNFP II: Lacto-N-fucopentaose II
LNFP III: Lacto-N-fucopentaose III
LNH: Lacto-N-hexaose
LNT: Lacto-N-tetraose
LNnT: Lacto-N-neotetraose
LSTb: Sialyl-lacto-N-tetraose b
LSTc: Sialyl-lacto-N-tetraose c
LacNac: N-Acetyl-D-Lactosamine
GH: Glycoside hydrolase
GEN: Gentamicin
KAN: Kanamycin
STREP: Streptomycin
TET: Tetracycline
ERY: Erythromycin
CHLORA: Chloramphenicol
AMP: Ampicillin
VAN: Vancomycin
