# Supplementary material for: Human milk oligosaccharide metabolism and antibiotic resistance in early gut colonizers: insights from bifidobacteria and lactobacilli in the maternal-infant microbiome
Source: Gut Microbes. 2025 May 9;17(1):2501192. doi: 10.1080/19490976.2025.2501192 (PMC12068340; doi:10.1080/19490976.2025.2501192)
Supplement: Supplemental Material [file KGMI_A_2501192_SM6649.zip › 7_Supl_Table7_bif_MIC.docx]

**Table 7.** *Bifidobacterium* spp phenotypic antibiotic resistance and MIC (µg/mL).

|  | | **GEN** | | **STREP** | **TET** | | **ERY** | | **CHLORA** | | **AMP** | | **VAN** | |  |
| --- | --- | --- | --- | --- | --- | --- | --- | --- | --- | --- | --- | --- | --- | --- | --- |
| ***B. adolescentis*** | | | | | | | | | | | | | | | |
|  | **IATA066** | | 256 | S | | S | | 16 | | S | | 8 | | S |  |
|  | **IATA096** | | S | 512 | | 64 | | 16 | | S | | 8 | | S |  |
|  | **IATA101** | | R | S | | S | | R | | S | | S | | S |  |
| ***B. animalis subps. lactis*** | | | | | | | | | | | | | | | |
|  | **IATA008** | | 256 | 256 | | 64 | | 16 | | S | | 16 | | S |  |
|  | **IATA010** | | 256 | S | | 32 | | 16 | | S | | 32 | | S |  |
|  | **IATA020** | | 256 | S | | 32 | | 16 | | S | | 32 | | S |  |
|  | **IATA021** | | 256 | 256 | | 64 | | 16 | | S | | S | | S |  |
|  | **IATA029** | | 256 | S | | 0.5 | | 16 | | S | | S | | S |  |
|  | **IATA135** | | 512 | 512 | | 64 | | 16 | | S | | 32 | | S |  |
|  | **IATA142** | | 512 | 256 | | 64 | | 16 | | S | | 8 | | S |  |
|  | **IATA143** | | 512 | 256 | | 64 | | 16 | | S | | 8 | | S |  |
| ***B. bifidum*** | | | | | | | | | | | | | | | |
|  | **IATA001** | | 512 | S | | 1 | | 16 | | S | | 4 | | S |  |
|  | **IATA005** | | 512 | S | | S | | 16 | | S | | S | | S |  |
|  | **IATA016** | | 256 | S | | S | | 16 | | S | | 4 | | S |  |
|  | **IATA039** | | 256 | 64 | | 0.5 | | 16 | | R | | 4 | | S |  |
|  | **IATA049** | |  |  | |  | |  | |  | |  | | S |  |
|  | **IATA102** | | 256 | S | | S | | 16 | | S | | 2 | | S |  |
|  | **IATA139** | | S | S | | S | | 16 | | S | | 2 | | S |  |
|  | **IATA148** | | 256 | 512 | | 32 | | 16 | | R | | 16 | | S |  |
| ***B. breve*** | | | | | | | | | | | | | | | |
|  | **IATA027** | |  |  | |  | |  | |  | |  | |  |  |
|  | **IATA048** | | S | 2048 | | 64 | | 16 | | S | | 8 | | S |  |
|  | **IATA077** | | S | 2048 | | 64 | | 16 | | S | | 4 | | S |  |
|  | **IATA084** | | 256 | S | | S | | 16 | | S | | 32 | | S |  |
|  | **IATA131** | | 256 | 2048 | | S | | 16 | | S | | 32 | | S |  |
|  | **IATA136** | | 256 | 2048 | | S | | 16 | | S | | 32 | | S |  |
|  | **IATA153** | | 256 | 1024 | | 64 | | 16 | | S | | 8 | | S |  |
| ***B. longum subsp. infantis*** | | | | | | | | | | | | | | | |
|  | **IATA104** | | 32 | S | | 32 | | 16 | | S | | 8 | | S |  |
|  | **IATA105** | | 32 | S | | 32 | | 16 | | S | | 4 | | S |  |
| ***B. longum subps. longum*** | | | | | | | | | | | | | | | |
|  | **IATA003** | | S | S | | S | | 16 | | S | | 16 | | S |  |
|  | **IATA015** | | R | S | | S | | 16 | | S | | 16 | | S |  |
|  | **IATA033** | | 128 | 64 | | 0.5 | | 16 | | R | | 32 | | S |  |
|  | **IATA034** | | S | S | | S | | 16 | | S | | 32 | | S |  |
|  | **IATA062** | | 128 | S | | S | | 16 | | S | | 32 | | S |  |
|  | **IATA075** | | 256 | S | | S | | 16 | | S | | 32 | | S |  |
|  | **IATA107** | | 128 | S | | S | | 16 | | S | | 32 | | S |  |
|  | **IATA116** | | 256 | S | | S | | 16 | | S | | 32 | | S |  |
|  | **IATA144** | | 128 | S | | 2 | | 16 | | S | | 32 | | S |  |
| ***B. pseudocatenulatum*** | | | | | | | | | | | | | | | |
|  | **IATA044** | | 256 | 128 | | 64 | | 8 | | R | | 32 | | S |  |
|  | **IATA160** | | 32 | 64 | | 1 | | 16 | | R | | 32 | | S |  |

GEN: gentamycin, STREP: streptomycin; TET: tetracycline; ERY: erythromycin; CHLORA: chloramphenicol; AMP: ampicillin; VAN: vancomycin.
