# Supplementary material for: Human milk oligosaccharide metabolism and antibiotic resistance in early gut colonizers: insights from bifidobacteria and lactobacilli in the maternal-infant microbiome
Source: Gut Microbes. 2025 May 9;17(1):2501192. doi: 10.1080/19490976.2025.2501192 (PMC12068340; doi:10.1080/19490976.2025.2501192)
Supplement: Supplemental Material [file KGMI_A_2501192_SM6649.zip › Supplementary Note 2.docx]

**Supplementary Note 2**. Genotype-to-phenotype predictions of bacterial HMO utilization.

All *B. adolescentis* strains were predicted to utilize lactose (Lac) but not any of the HMO structures due to the absence of HMO transporters and most glycoside hydrolases (GHs) required for HMO metabolism (**Figure 3a**, **Supplementary Table 2**). In agreement with *in silico* predictions, *B. adolescentis* IATA066, IATA096, and IATA101 did not utilize any HMOs (**Figure 3b**).

Similarly, all *B. animalis* subsp. *lactis* (*B. lactis*) strains were predicted to utilize Lac but not any HMO species (**Figure 3a**, **Supplementary Table 2**). In agreement with *in silico* predictions, strains IATA008, IATA021, IATA135, IATA142, and IATA143 did not utilize any HMOs (**Figure 3b**). However, there was a discrepancy for strains IATA010, IATA020, and IATA029, which depleted lacto-*N*-tetraose (LNT) from the pHMO mixture. These strains also grew in a medium supplemented with LNT as the sole carbon source (**Supplementary Figure 4a**). Given the very high genomic similarity of tested *B. lactis* strains, additional studies are required to explain the observed discrepancy.

*B. pseudocatenulatum* strains were predicted to utilize Lac, lacto-*N*-biose (LNB), and LNT intracellularly (**Figure 3a**, **Supplementary Figure 2**). In agreement with *in silico* predictions, all strains consumed over 95% of LNT from the pHMO mixture (**Figure 3b**).

All *B. breve* strains were predicted to utilize Lac, LNB, LNT, and lacto-*N*-neotetraose (LNnT) via intracellular catabolic pathways (**Figure 3a**, **Supplementary Figure 2**). In agreement with *in silico* predictions, *B. breve* strains IATA027 and IATA153 efficiently consumed both LNT (>90%) and LNnT (>75%) (**Figure 3b**). The remaining *B. breve* strains depleted only LNT. Consistent with glycoprofining data, *B. breve* IATA048 and IATA131 grew in a medium supplemented with LNT but not LNnT (**Supplementary Figure 4a**). The discrepancy between genomic predictions and experimental data about LNnT utilization could be potentially linked to insufficient induction or mutations in genes involved in the metabolism of this oligosaccharide. For example, the *nahS* gene in *B. breve* IATA131 was disrupted due to a premature stop codon, suggesting that the encoded substrate-binding component of the LNnT-specific ABC transporter^1^ might be nonfunctional (**Supplementary Figure 3**).

All *B. bifidum* strains possessed extracellular GHs with diverse specificities, potentially enabling them to completely or partially degrade all tested HMOs and utilize released Lac and LNB (**Figure 3a**, **Supplementary Figure 2**). In agreement with this notion, all *B. bifidum* strains depleted most HMO species from the pHMO mixture (**Figure 3b**). The depletion of 2’-fucosyllactose (2'FL) by all strains, except IATA039, was consistent with the presence of an extracellular α-fucosidase (BbAfcA; GH95) that releases terminal α-1,2-linked fucosyl residues from fucosylated HMOs^2^ (**Supplementary Table 2**). The *bbAfcA* gene in *B. bifidum* IATA039 was truncated at the 5’-end, resulting in a 151 amino acid shorter protein, which was likely nonfunctional due to the removal of the N-terminal signal peptide (**Supplementary Figure 3**). Thus, the *bbAfcA* gene truncation could explain the strain’s inability to utilize 2’FL as well as the decreased degradation of lacto-*N*-fucopentaose I (LNFP I).

We observed an interplay between concentrations of 3-fucosyllactose (3FL) and difucosyllactose (DFLac) in the supernatants of all strains except IATA049 and IATA039 (**Figure 3b**). The increased 3FL concentration could be explained by the unequal (separated in time) removal of fucosyl residues from DFLac by the α-fucosidases of *B. bifidum*. Specifically, the terminal α-1,2-fucosyl residues would be removed first by BbAfcA, generating 3FL and thus temporally increasing its concentration. A similar interplay likely occurred between concentrations of sialyl-lacto-*N*-tetraose c (LSTc) and disialylated HMO species, given the increase of LSTc concentration in supernatants of all *B. bifidum* strains. For example, extracellular α-sialidases SiaBB1 and SiaBB2 would remove a single terminal *N*-acetylneuraminic acid residue from disialylated HMOs generating LSTc.

In agreement with *in silico* predictions, all *B. bifidum* strains efficiently (>90%) depleted the LNT in the pHMO mixture (**Figure 3a,b**). In contrast, the concentration of LNnT in the supernatants of *B. bifidum* strains varied considerably. It decreased in the case of IATA139 and IATA148, remained relatively unchanged in the supernatants of five strains (IATA005, IATA039, IATA049, IATA102, IATA123), and increased in supernatants of IATA001 and IATA016 strains. Since LNnT is an intermediate product of the degradation of multiple more complex HMOs, the observed results may reflect strain-level variability in the kinetics of long-chain HMO utilization. *B. bifidum* strains IATA001, IATA005, IATA049, and IATA102 were able to grow on LNnT as a sole carbon source, confirming their capacity to utilize this oligosaccharide (**Supplementary Figure 4a**).

All *B. bifidum* strains were predicted to degrade 3’- and 6’-siallylactoses (3’SL and 6’SL) by extracellular α-sialidases SiaBB1 and SiaBB2 (GH33)^3,4^ (**Figure 3a**, **Supplementary Figure 2**). However, only 6’SL was partially degraded for all strains except IATA148, whereas the depletion of 3’SL was strain-specific (**Figure 3b**). In agreement with predictions, all *B. bifidum* strains degraded various lcHMOs, e.g., lacto-*N*-fucopentaoses II/III (LNFP II/III), disialyllacto-*N*-tetraose (DSLNT), fucosyllacto-*N*-hexaose (FLNH), difucosyllacto-*N*-hexaose (DFLNH), and disialyllacto-N-hexaose (DSLNH).

*B. longum* subsp. *infantis* (*B. infantis*) strains were predicted to utilize Lac, LNB, LNT, LNnT, 2’FL, 3’FL, DFLac, LNFP I, 3’SL, 6’SL as well as lcHMOs intracellularly (**Figure 3a**, **Supplementary Figure 2**). In agreement with *in silico* predictions, all strains utilized 2’FL, 3’FL, LNT, and LNFP I, and certain lcHMOs (LNFP II and DFLNT). In contrast, *B. infantis* IATA104 and IATA105 did not utilize DFLac and 3’SL/6’SL, and the IATA105 strain did not utilize LNnT. *B. infantis* IATA105 also did not grow in a medium supplement with LNnT (**Supplementary Figure 4a**). Low DFLac and 3’SL/6’SL consumption could be related to the preferential utilization of HMO species from the pHMO mixture. The absence of LNnT utilization by *B. infantis* IATA105 was potentially due to mutations in genes constituting the respective catabolic pathway.

All *B. longum* subsp. *longum* (*B. longum*) strains were predicted to utilize Lac and LNB (**Figure 3a**). Strains IATA034, IATA062, and IATA075 possessed an extracellular lacto-*N*-biosidase (LnbX; GH136; **Supplementary Table 2**). This enzyme has a broad substrate specificity and can cleave the internal -GlcNA(c(b1-3)Gal- glycosidic bond in various HMOs, including LNT, LNFP I, and lacto-*N*-hexaose (LNH)^5^. Thus, these three *B. longum* strains were predicted to extracellularly degrade LNT (to LNB and Lac) and LNFP I (to H1 trisaccharide and Lac; **Supplementary Figure 2**). In agreement with *in silico* predictions, all three strains completely (>95%) depleted LNT from the pHMO mixture, and two strains (IATA062 and IATA034) degraded 30-50% of LNFP I (**Figure 3b**). The partial hydrolysis by LnbX might also explain the depletion of LNH and FLNH in the supernatants of *B. longum* IATA034, IATA062, and IATA075.

*B. longum* IATA116 was predicted to utilize LNT intracellularly and, in agreement with the predictions, consumed over 95% of this HMO species (**Figure 3a,b**). The remaining *B. longum* strains (IATA003, IATA015, IATA144, IATA033, IATA107) were predicted not to utilize LNT because their variant of the substrate-binding component (GltA) of the LNB/GNB/LNT transporter (GltABC) was previously shown to have low affinity for LNT^6^ . However, only *B. longum* IATA144 did not consume LNT, whereas the remaining three strains efficiently depleted it. Consistent with glycoprofiling data, *B. longum* IATA003, IATA015, IATA033, and IATA107 grew in a medium supplemented on LNT as the sole carbon source (**Supplementary Figure 4a**). Additional studies on the substrate-specificity of different GltA orthologs are required to explain the observed discrepancies.

The decrease of LNnT and LNFP III concentration in supernatants of certain strains (e.g., IATA034) was likely not due to the intracellular utilization of these HMOs but rather partial extracellular degradation by a β-1,4-galactosidase which would cleave the terminal galactosyl residue from LNnT (leaving lacto-*N*-triose II) and LNFP III (leaving fucosyllacto-*N*-triose II). Consistent with this hypothesis, *B. longum* IATA034 did not grow on LNnT as the sole carbon source (**Supplementary Figure 4a**).

Reconstruction of 11 carbohydrate utilization pathways suggested that all lactobacilli could utilize specific structural components of HMOs, namely Lac, *N*-acetylglucosamine (GlcNAc), galactose, and glucose (**Figure 4a**, **Supplementary Table 3**). However, the predicted abilities to metabolize LNnT, lacto-*N*-triose II (LNTriose), *N*-acetyllactosmine (LacNAc), LNB, fucose, and *N*-acetylneuraminic acid were species- and strain-specific.

Only *Limosilactobacillus mucosae* IATA082, *Lacticaseibacillus rhamnosus* IATA115, and *Latilactobacillus sakei* IATA088 genomes encode the two-subunit extracytoplasmic β-galactosidase LacLM that cleaves the terminal non-reducing galactosyl residue from LNnT, releasing LNTriose^7^ (**Figure 4b**). However, these genomes lacked the *bnaG* gene encoding a secreted β-*N*-acetylglucosaminidase that releases a GlcNAc residue from LNTriose^8^ . Therefore, the proposed LNnT utilization mechanism for these strains involved the partial extracellular degradation of LNnT and the uptake of released galactose. Consistent with this prediction, *L. mucosae* IATA082 and *L. sakei* IATA088 depleted LNnT from the pHMO mixture and grew in the medium supplemented with LNnT as the sole carbon source (**Figure 4c**, **Supplementary Figure 4b**).

None of the 14 lactobacilli strains were predicted to metabolize LNT, which was in agreement with glycoprofiling data for all strains except *L. mucosae* IATA082. In addition to LNT, the later strain partially depleted LNFP I/III, LNH, DFLNH, and FNLH from the culture medium. Given that the genome of *L. mucosae* IATA082 lacks genes encoding GH29 and GH95 α-fucosidases (**Supplementary Table 3**), the decrease in concentrations of these HMOs could be potentially linked to partial degradation via the removal of terminal non-reducing galactosyl resides by β-galactosidases. However, further studies are required to test this hypothesis.

**REFERENCES**

1. James K, Motherway MO, Bottacini F, van Sinderen D. Bifidobacterium breve UCC2003 metabolises the human milk oligosaccharides lacto-N-tetraose and lacto-N-neo-tetraose through overlapping, yet distinct pathways. Sci Rep 2016; 6:38560.

2. Katayama T, Sakuma A, Kimura T, Makimura Y, Hiratake J, Sakata K, Yamanoi T, Kumagai H, Yamamoto K. Molecular cloning and characterization of Bifidobacterium bifidum 1,2-alpha-L-fucosidase (AfcA), a novel inverting glycosidase (glycoside hydrolase family 95). J Bacteriol 2004; 186:4885–93.

3. Nishiyama K, Yamamoto Y, Sugiyama M, Takaki T, Urashima T, Fukiya S, Yokota A, Okada N, Mukai T. Bifidobacterium bifidum Extracellular Sialidase Enhances Adhesion to the Mucosal Surface and Supports Carbohydrate Assimilation. mBio 2017; 8:e00928-17.

4. Kiyohara M, Tanigawa K, Chaiwangsri T, Katayama T, Ashida H, Yamamoto K. An exo-alpha-sialidase from bifidobacteria involved in the degradation of sialyloligosaccharides in human milk and intestinal glycoconjugates. Glycobiology 2011; 21:437–47.

5. Sakurama H, Kiyohara M, Wada J, Honda Y, Yamaguchi M, Fukiya S, Yokota A, Ashida H, Kumagai H, Kitaoka M, et al. Lacto-N-biosidase encoded by a novel gene of Bifidobacterium longum subspecies longum shows unique substrate specificity and requires a designated chaperone for its active expression. J Biol Chem 2013; 288:25194–206.

6. Suzuki R, Wada J, Katayama T, Fushinobu S, Wakagi T, Shoun H, Sugimoto H, Tanaka A, Kumagai H, Ashida H, et al. Structural and Thermodynamic Analyses of Solute-binding Protein from Bifidobacterium longum Specific for Core 1 Disaccharide and Lacto-N-biose I *. J Biol Chem 2008; 283:13165–73.

7. Thongaram T, Hoeflinger JL, Chow J, Miller MJ. Human milk oligosaccharide consumption by probiotic and human-associated bifidobacteria and lactobacilli. J Dairy Sci 2017; 100:7825–33.

8. Bidart GN, Rodríguez-Díaz J, Palomino-Schätzlein M, Monedero V, Yebra MJ. Human milk and mucosal lacto- and galacto-N-biose synthesis by transgalactosylation and their prebiotic potential in Lactobacillus species. Appl Microbiol Biotechnol 2017; 101:205–15.
