# Supplementary material for: Human milk oligosaccharide metabolism and antibiotic resistance in early gut colonizers: insights from bifidobacteria and lactobacilli in the maternal-infant microbiome
Source: Gut Microbes. 2025 May 9;17(1):2501192. doi: 10.1080/19490976.2025.2501192 (PMC12068340; doi:10.1080/19490976.2025.2501192)
Supplement: Supplemental Material [file KGMI_A_2501192_SM6649.zip › Supplementary Note 3.docx]

**Supplementary Note 3**. Antibiotic resistance phenotype of bifidobacteria and lactobacilli.

All *Bifidobacterium* strains exhibited resistance to erythromycin but were susceptible to chloramphenicol and vancomycin.

*Bifidobacterium bifidum:*

Among the *Bifidobacterium bifidum* strains, strain IATA139 is the only one susceptible to gentamicin, whereas IATA001 and IATA005 exhibited the highest resistance to gentamicin, with MIC values reaching 512 µg/mL. For ampicillin, IATA148 exhibited the highest MIC (16 µg/mL), IATA005 was the only strain susceptible, and the remaining strains showed moderate resistance (MIC = 2-4 µg/mL). IATA039 and IATA148 were the only strains resistant to streptomycin (64 and 512 µg/mL, respectively) and tetracycline (0.5 and 32 µg/mL, respectively).

*Bifidobacterium longum subsp. longum:*

In the *Bifidobacterium longum subsp. longum* group, strain IATA033 was susceptible to most antibiotics. Other strains, such as IATA015, IATA062, IATA075, IATA107, IATA116, and IATA137, were resistant to gentamicin and ampicillin. Interestingly, resistance to tetracycline was observed in IATA033 and IATA144, but not in IATA075.

*Bifidobacterium longum subsp. infantis:*

*Bifidobacterium longum subsp. infantis* strains had a similar resistance profile: they showed resistance to gentamicin (MIC = 32 µg/mL) and ampicillin (MIC = 4-8 µg/mL). Strain IATA045 was the only one resistant to streptomycin.

*Bifidobacterium animalis subsp. lactis:*

*Bifidobacterium animalis* strains exhibited high resistance to gentamicin (MIC = 256-512 µg/mL). Strain IATA029, exhibited lower resistance levels (32 µg/mL for tetracycline) compared to other strains in this subspecies, indicating potential variations in resistance mechanisms or gene expression levels. IATA021 and IATA029 were the only strains susceptible to ampicillin, while IATA142 and IATA143 had MICs of 8 µg/mL, IATA010 and IATA020 had MICs of 32 µg/mL, and IATA008 had an MIC of 16 µg/mL. All strains, except IATA010, IATA020, and IATA029, showed high resistance to streptomycin (MIC = 256-512 µg/mL).

*Bifidobacterium breve:*

*Bifidobacterium breve* strains showed significant variability in their resistance profiles. For instance, IATA048 and IATA077 exhibited extremely high resistance to streptomycin (MIC = 2048 µg/mL). In contrast, IATA084 remained susceptible to streptomycin and tetracycline but was resistant to gentamicin (MIC = 256 µg/mL). IATA131, IATA136, and IATA153 showed resistance to gentamicin (MIC = 256 µg/mL) and streptomycin (MIC = 1024-2048 µg/mL), with IATA153 also resistant to tetracycline.

*Bifidobacterium adolescentis:*

Bifidobacterium adolescentis strains generally showed low resistance levels, with strains like IATA066 showing resistance to gentamicin (MIC = 256 µg/mL) and ampicillin (MIC = 8-16 µg/mL). However, IATA101 stood out with resistance to streptomycin, tetracycline, and ampicillin, in addition to the already mentioned erythromycin.

*Lactobacillus*

All Lactobacillus strains showed susceptibility to the antibiotic chloramphenicol. Regarding *Lactobacillus paragasseri,* all strains were resistant to erythromycin (MIC = 16 µg/mL) and ampicillin, with IATA126 being the least resistant to ampicillin (MIC = 2 µg/mL). However, this strain was the only one resistant to gentamicin (MIC = 512 µg/mL) among the L. paragasseri strains. Additionally, IATA108 exhibited resistance to streptomycin (MIC = 1024 µg/mL).

For *L. gasseri,* both strains (IATA081 and IATA122) displayed the same resistance profile: they were resistant to gentamicin (MIC = 128 µg/mL), kanamycin (MIC = 1024 µg/mL), erythromycin (MIC = 16 µg/mL), and ampicillin (MIC = 8-16 µg/mL), as well as resistant to streptomycin, tetracycline, and chloramphenicol.

*Lacticaseibacillus paracasei* IATA110 was resistant to most of the antibiotics tested, while IATA109 was only resistant to erythromycin (MIC = 16 µg/mL) and ampicillin (MIC = 32 µg/mL). The strain IATA083 was the only one susceptible to all antibiotics, and no antibiotic resistance genes were found in its genome.

Similarly, *L. rhamnosus* IATA117 was susceptible to all antibiotics as well. IATA116 was resistant to erythromycin (MIC = 16 µg/mL) and ampicillin (MIC = 32 µg/mL) but susceptible to tetracycline, despite the presence of the tetM gene in its genome.

The unique strain of *L. sakei* (IATA088) showed resistance to all antibiotics except erythromycin and chloramphenicol. The same phenotype was observed in *L. mucosae* IATA081, which carries the lincosamide resistance gene lnuC. Finally, *Ligilactobacillus ruminis* IATA127 exhibited high resistance to gentamicin (MIC = 512 µg/mL), kanamycin (MIC = 1024 µg/mL), streptomycin (MIC = 512 µg/mL), tetracycline (MIC = 64 µg/mL), erythromycin (MIC = 16 µg/mL), and ampicillin (MIC = 32 µg/mL), but was susceptible to chloramphenicol, as were all other *Lactobacillus* strains.
