# Supplementary material for: Human milk oligosaccharide metabolism and antibiotic resistance in early gut colonizers: insights from bifidobacteria and lactobacilli in the maternal-infant microbiome
Source: Gut Microbes. 2025 May 9;17(1):2501192. doi: 10.1080/19490976.2025.2501192 (PMC12068340; doi:10.1080/19490976.2025.2501192)
Supplement: Supplemental Material [file KGMI_A_2501192_SM6649.zip › 8_Supl_Table8_lacto_MIC.docx]

**Table 8.** Lactobacilli phenotypic antibiotic resistance and MIC (µg/mL).

|  |  | **GEN** | **KAN** | **STREP** | **TET** | **ERY** | **CHLORA** | **AMP** |
| --- | --- | --- | --- | --- | --- | --- | --- | --- |
| ***L. paragasseri*** | | | | | | | | |
|  | **IATA108** | S | S | 1024 | S | 16 | S | 32 |
|  | **IATA126** | 512 | S | S | S | 16 | S | 2 |
|  | **IATA140** | S | S | S | S | 16 | S | 16 |
|  | **IATA141** | S | S | S | S | 16 | S | 16 |
| ***L. gasseri*** | | | | | | | | |
|  | **IATA081** | 128 | 1024 | S | S | 16 | S | 8 |
|  | **IATA122** | 128 | 1024 | S | S | 16 | S | 16 |
| ***L. paracasei*** | | | | | | | | |
|  | **IATA109** | S | S | S | S | 16 | S | 32 |
|  | **IATA083** | S | S | S | S | S | S | S |
|  | **IATA110** | 256 | 1024 | 256 | S | 16 | S | 32 |
| ***L. rhamnosus*** | | | | | | | | |
|  | **IATA115** | S | S | S | S | 16 | S | 32 |
|  | **IATA117** | S | S | S | S | S | S | S |
| ***L. sakei*** | | | | | | | | |
|  | **IATA088** | 32 | 128 | 128 | 8 | S | S | 32 |
| ***L. mucosae*** | | | | | | | | |
|  | **IATA082** | 16 | 128 | 64 | 4 | S | S | 32 |
| ***L. ruminis*** | | | | | | | | |
|  | **IATA127** | 512 | 1024 | 512 | 64 | 16 | S | 32 |

GEN: gentamycin, KAN: kanamycin; STREP: streptomycin; TET: tetracycline; ERY: erythromycin; CHLORA: chloramphenicol; AMP: ampicilin
