# Supplementary material for: Human milk oligosaccharide metabolism and antibiotic resistance in early gut colonizers: insights from bifidobacteria and lactobacilli in the maternal-infant microbiome
Source: Gut Microbes. 2025 May 9;17(1):2501192. doi: 10.1080/19490976.2025.2501192 (PMC12068340; doi:10.1080/19490976.2025.2501192)
Supplement: Supplemental Material [file KGMI_A_2501192_SM6649.zip › Supplementary Note 1.docx]

**Supplementary Note 1**. Modified peptone-yeast-glucose (mPYG) medium reagents and preparation.

Solution 1: combine the following and stir at room temperature:

| **Reagent** | **Supplier** | **Cat. No.** | **Concentration** |
| --- | --- | --- | --- |
| Tryptone | Teknova | T9011 | 5 g/L |
| BBL Biosate Peptone | BD | 211862 | 5 g/L |
| Yeast Extract |  | Y1003-1001 | 10 g/L |
| Beef Extract Powder | Sigma-Aldrich | B4888-50G | 5 g/L |
| Dextrose (anhydrous) | Millipore | DX0145-1 | 5 g/L |
| K_2_HPO_4_ | Sigma-Aldrich | P3786-500G | 2 g/L |
| Cysteine-HCl | Sigma-Aldrich | C1276-10G | 0.5 g/L |

Solution 2 (salt solution): Combine the following and stir until all reagents are dissolved. Filter sterilize and store at room temperature:

| **Reagent** | **Supplier** | **Cat. No.** | **Quantity** |
| --- | --- | --- | --- |
| CaCl_2_ x 2 H_2_O | Sigma-Aldrich | C5080-500G | 125 mg/L |
| MgSO_4_ x 7 H_2_O | Sigma-Aldrich | M5921-500G | 250 mg/L |
| K_2_HPO_4_ | Sigma-Aldrich | P3786-500G | 0.50 g/L |
| KH_2_PO_4_ | Sigma-Aldrich | P0662-500G | 0.50 g/L |
| NaHCO_3_ | Sigma-Aldrich | S8875-500G | 5.00 g/L |
| NaCl | Fisher Scientific | BP358-212 | 1.00 g/L |

Solution 3 (haemin solution): Combine the following in a 15 mL conical and vortex to dissolve. Filter sterilize into a new 15 mL conical tube and store at 4C wrapped in foil.

| **Reagent** | **Supplier** | **Cat. No.** | **Quantity** |
| --- | --- | --- | --- |
| Haemin chloride | Sigma-Aldrich | 3741 | 50 mg/L |
| 1 N NaOH | Sigma-Aldrich | S5881-500G | 0.1% |

Solution 4 (vitamin k solution): Combine the following in a 15 mL conical and vortex to dissolve. Filter sterilize into a new 15 mL conical tube and store at 4ºC wrapped in foil.

| **Reagent** | **Supplier** | **Cat. No.** | **Quantity** |
| --- | --- | --- | --- |
| Vitamin K1 | Thermo Scientific | L10575.06 | 100 uL |
| 95% EtOH | Koptec | V1101 | 20 mL |

Add the following volumes of Solutions 2-4 to Solution 1.

| **Solution** | **volume** |
| --- | --- |
| Solution 2 (salt solution) | 120 mL |
| Solution 3 (haemin solution) | 1 mL |
| Solution 4 (Vitamin K solution) | 200 uL |

Filter sterilize combined medium in biosafety cabinet.

Store bottle(s) wrapped in foil at 4C.
