# Supplementary figures and images for: Human milk oligosaccharide metabolism and antibiotic resistance in early gut colonizers: insights from bifidobacteria and lactobacilli in the maternal-infant microbiome
Source: Gut Microbes. 2025 May 9;17(1):2501192. doi: 10.1080/19490976.2025.2501192 (PMC12068340; doi:10.1080/19490976.2025.2501192)

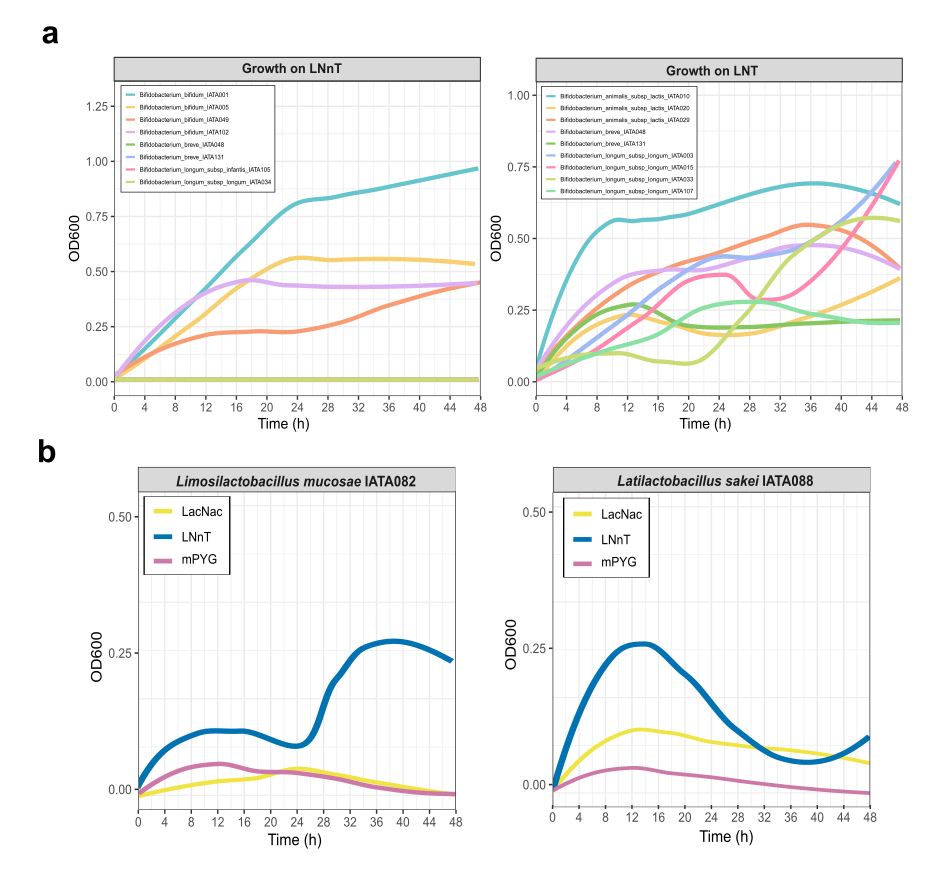

Supplement: Supplemental Material [file KGMI_A_2501192_SM6649.zip › Supl_figure_4_curves_bif_lac.png]

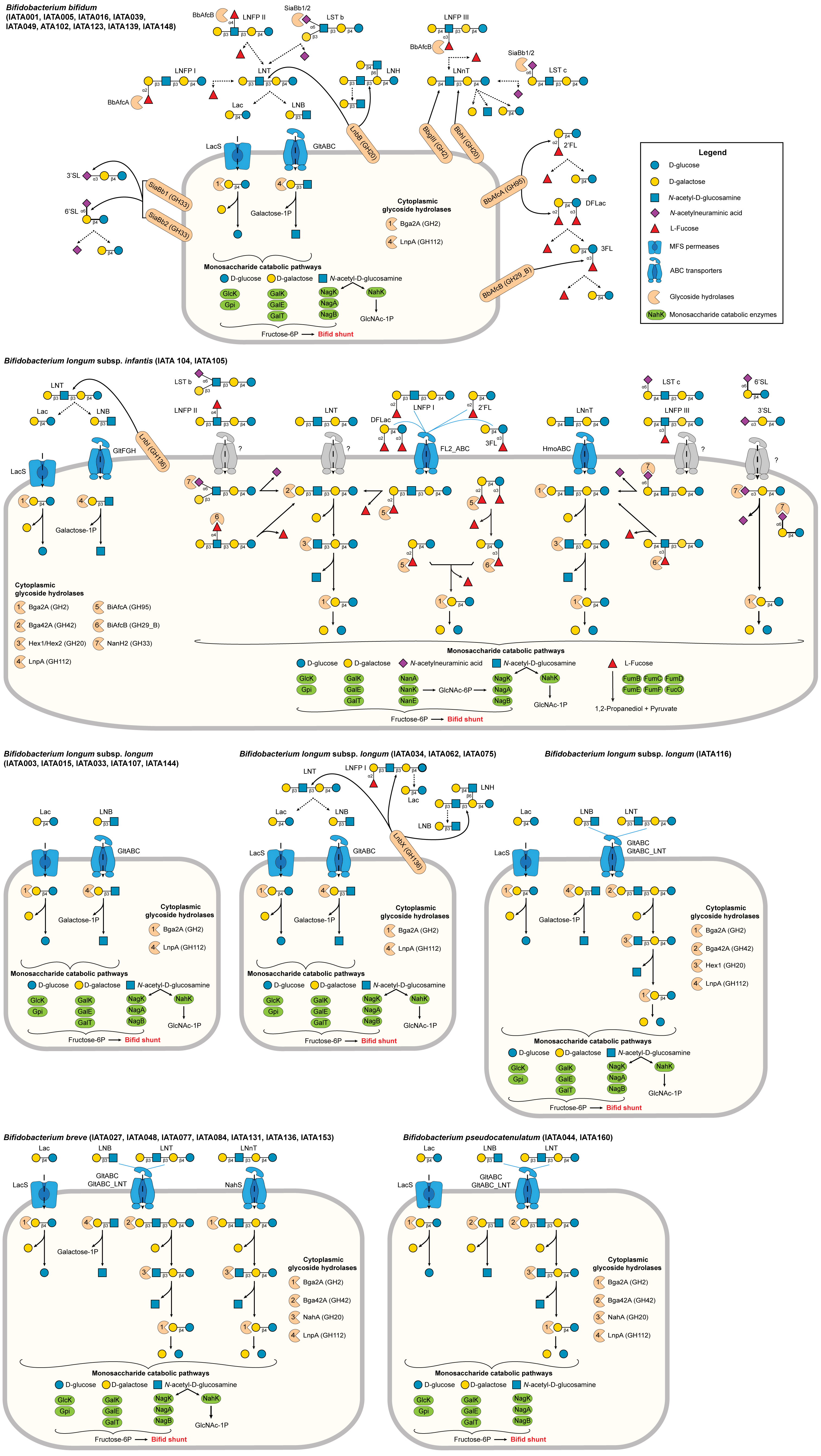

Supplement: Supplemental Material [file KGMI_A_2501192_SM6649.zip › Supl_figure_2_pathways.png]

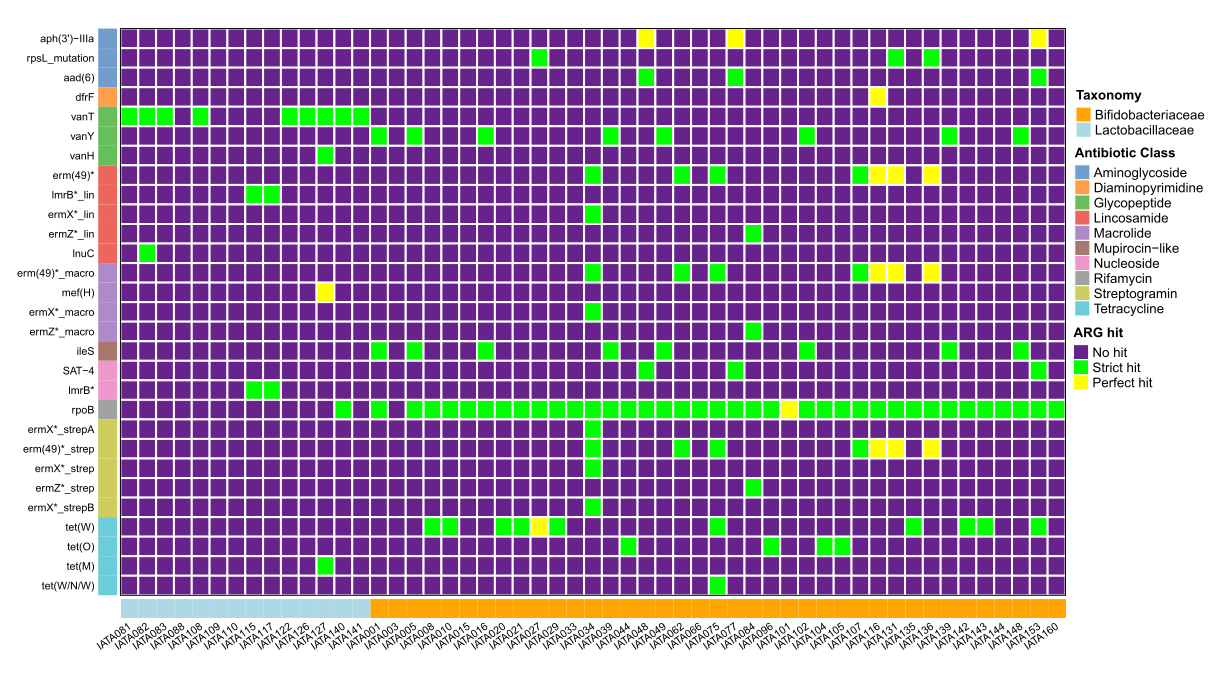

Supplement: Supplemental Material [file KGMI_A_2501192_SM6649.zip › Supl_figure_5_ARG_heatmap.png]

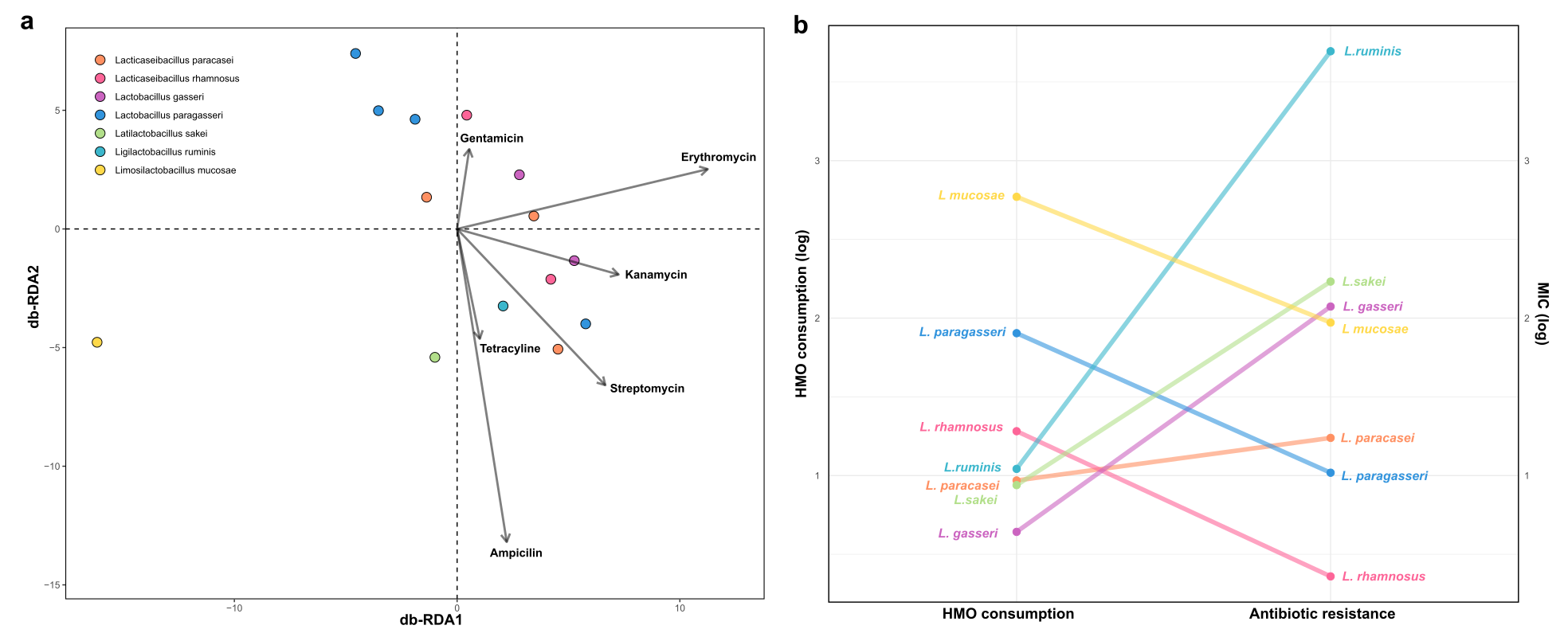

Supplement: Supplemental Material [file KGMI_A_2501192_SM6649.zip › Supl_figure_6_RDA_lactos.png]

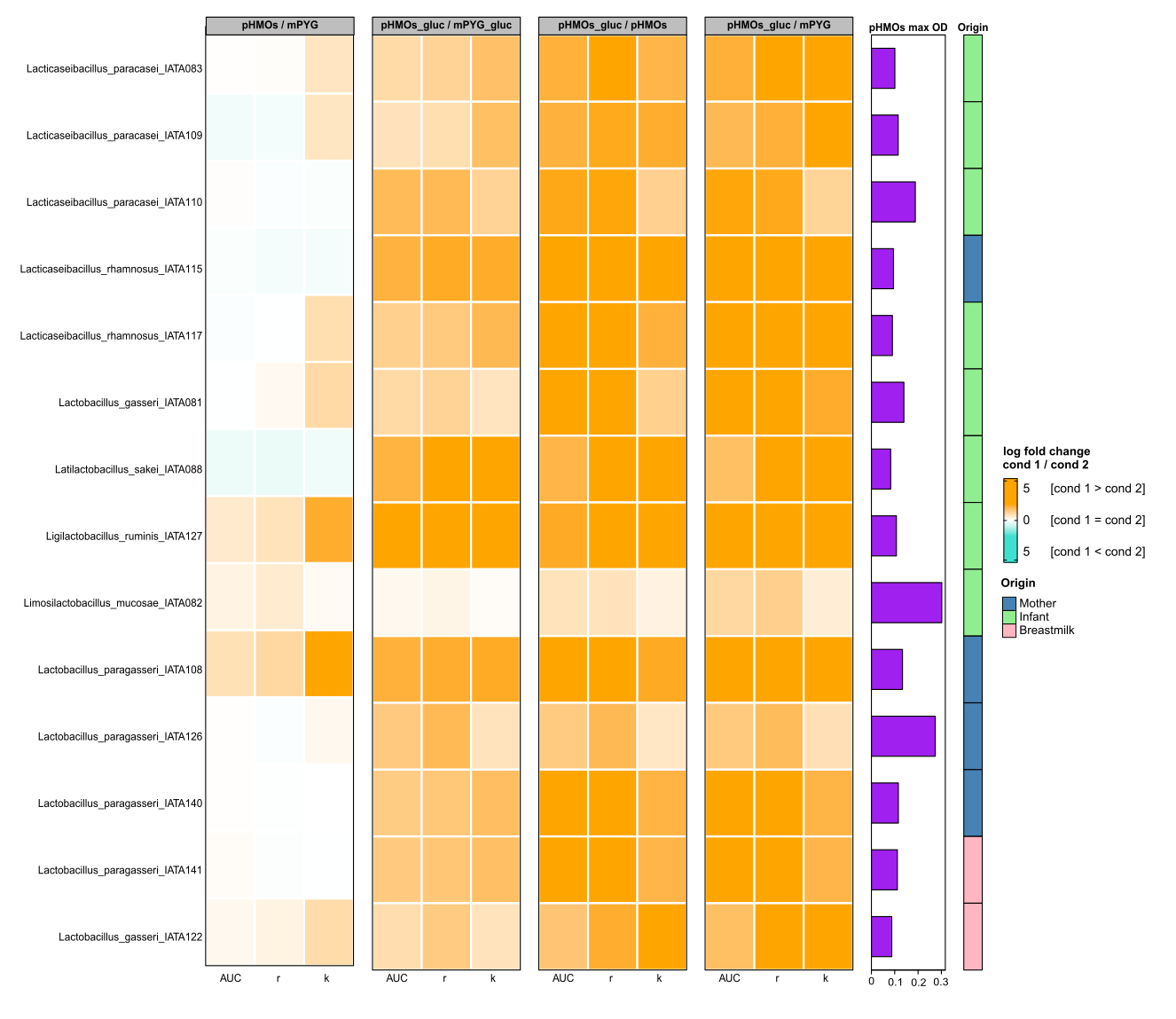

Supplement: Supplemental Material [file KGMI_A_2501192_SM6649.zip › Supl_figure_1_lacto_heatmap.png]

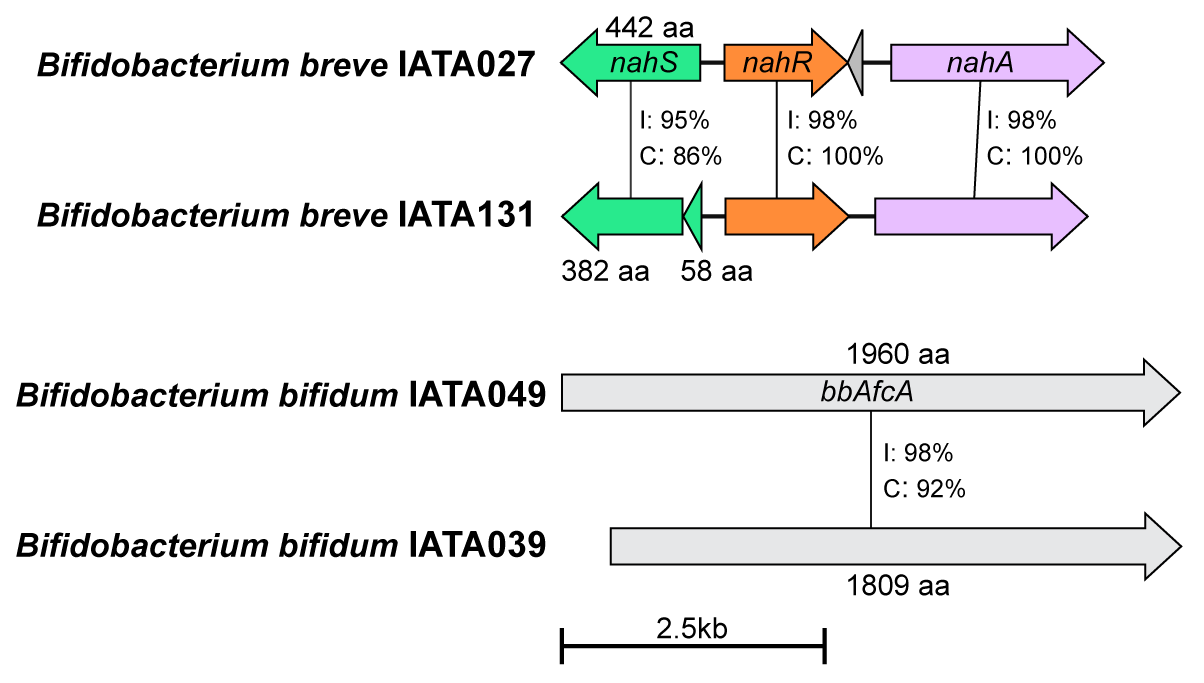

Supplement: Supplemental Material [file KGMI_A_2501192_SM6649.zip › Supl_figure_3_gene_clusters.png]
